# DIBALH: from known fundamental to an unusual reaction; chemoselective partial reduction of tertiary amides in the presence of esters†

**DOI:** 10.1039/d1ra06279d

**Published:** 2021-10-18

**Authors:** Yu Jin Heo, Hyun Tae Kim, Ashok Kumar Jaladi, Duk Keun An

**Affiliations:** Department of Chemistry, Institute for Molecular Science and FusionTechnology, Kangwon National University Chunchon 24341 Republic of Korea dkan@kangwon.ac.kr

## Abstract

This study presents a quick and reliable approach to the chemoselective partial reduction of tertiary amides to aldehydes in the presence of readily reducible ester groups using commercial DIBALH reagent. Moreover, the developed method was also extended to multi-functional molecules bearing ester moieties, which were successfully chemoselectively reduced to the corresponding aldehydes.

## Abstract 2

Highly chemoselective partial reduction of tertiary amides to aldehydes over esters are reported with commercial DIBALH. Various aldehydes are synthesized in excellent yields.

## Graphical abstract

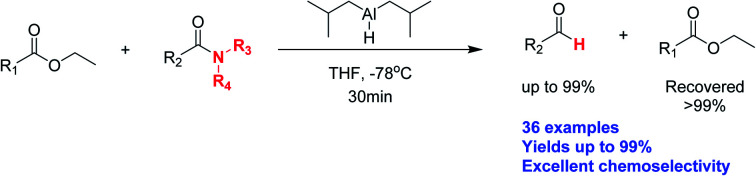

## Introduction

Chemoselectivity, the preferential transformation of one of two or more functional groups that are present in a molecule or reactant, can be a useful method for protecting-group-free synthesis of complex natural products or other materials.^1^ Although several reagents and catalysts have been reported as suitable for chemoselective functional group manipulations, the conversion of relatively less reactive functional groups in the presence of readily reacting ones is highly interesting and uncommon.

The partial reduction of amides to aldehydes^2,3^ in the presence of ester groups is one of the best examples for such transformations. Even though the reduction of amides to alcohols or amines has been known for several years,^4^ only a limited number of reagents have been hitherto reported for partial reduction of amides to aldehydes in the presence of esters.

For example, Hwu and co-workers^5^ reported on a system for selective reduction of aliphatic amides using alkyltrifluoromethanesulphonate and L-Selectride, giving the corresponding aldehydes in moderate yields. Recently, the Schwartz (Cp_2_Zr(H)Cl) reagent^6^ and *in situ*-prepared Schwartz reagents^7^ showed promising results in the selective reduction of amides to aldehydes. However, these reagents are highly expensive and sensitive to long-term storage.^6*c*–*e*^

A magnesium-based borohydride system was reported by Singaram and co-workers^8^ for the selective reduction of Weinreb amides. However, this system requires additional steps to obtain the desired aldehyde. More recently, Adolfsson and co-workers reported the use of a catalytic hydrosilylation system with TMDS/(Mo(CO)_6_) for the selective reduction of amides.^9^ Nevertheless, this reaction afforded a mixture of products from aliphatic amides containing α-hydrogens. Both of these reported systems involve highly expensive, sensitive reagents and possess practical difficulties such as limitations to either aromatic or aliphatic substrates. In order to overcome the difficulties encountered in previous methods, it is crucial to develop a more efficient and readily available reagent that would participate in the selective reduction of amides to aldehydes over esters.

**Scheme 1:**
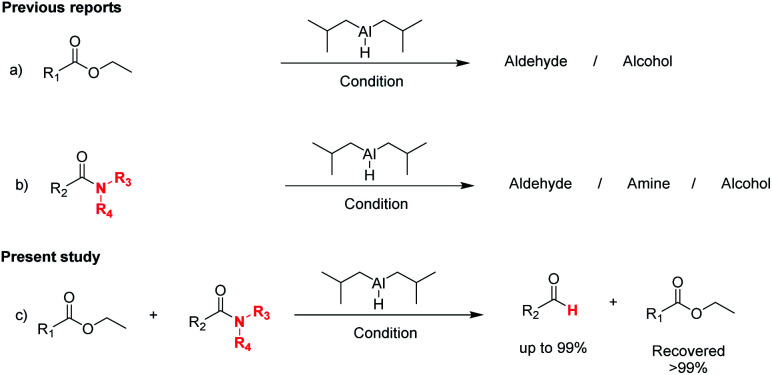
DIBALH mediated reductions of esters and tertiary amides.

The reduction of amides with DIBALH and reagents derived from DIBALH is a known reaction.^10^ Previous reports on the reduction of amides with DIBALH at 0 °C or room temperature resulted in a mixture of aldehydes, alcohols, and amines.^11^ Considering the information available on the modification of DIBALH and Red-Al reagents utilized in the partial reduction of various carbonyl and acid derivatives,^12^ in this study we observed the chemoselective reduction of tertiary amides such as Weinreb and morpholine amides to the corresponding aldehydes in the presence of esters with commercial DIBALH (Scheme 1).

## Results and discussion

Initially, we carried out a comparative study on the partial reduction of esters and tertiary amides with DIBALH in various solvents. The reactions were performed in THF, hexane, Et_2_O, CH_2_Cl_2_, toluene, and DME. While the amide reduction in hexane was almost inert (entry 5 in Table 1), the reaction in DME resulted in a good yield (76%) with high chemoselectivity (entry 9 in Table 1). Notably, the reaction in THF at −78 °C gave the aldehyde in almost quantitative yield (entry 1 in Table 1), while at −20 and 0 °C only 46% and 32% yields, respectively, were obtained (entries 2 and 4 in Table 1). Additionally, the reactions in ether and toluene afforded lower aldehyde yield with moderate chemoselectivity (entries 7 and 8 in Table 1).

**Table 1:** Reaction conditions for the chemoselective partial reduction of tertiary amides in the presence of esters

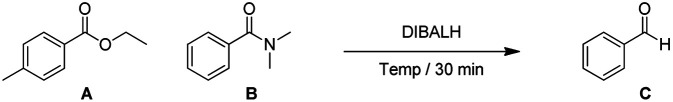					
Entry	DIBALH (eq.)	Temp (°C)	Solvent	Recovery of A^a^ (%)	Yield of C^a^ (%)
1	1.1	−78	THF	>99	>99
2	1.1	−20	THF	>99	46
3	2.1	−20	THF	41	47
4	1.1	0	THF	93	32
5	1.1	−78	Hexane	35	1
6	1.1	−78	CH_2_Cl_2_	69	17
7	1.1	−78	Et_2_O	85	49
8	1.1	−78	Toluene	63	34
9	1.1	−78	DME	>99	76

a Yields were determined by GC.

After successfully attaining the selective reduction of tertiary amides in the presence of esters in THF, we applied the respective conditions to the partial reduction of various *N*,*N*-dimethyl amides to the corresponding aldehydes. As shown in Table 2, *N*,*N*-dimethyl amides were reduced to aldehydes in the presence of ethyl benzoate. Aromatic compounds with electron-donating and withdrawing groups afforded excellent to quantitative yields of the corresponding aldehydes (entries 1–8 in Table 2). Polyaromatic compound, 2-naphthylamide, resulted in the partial reduction to aldehyde with quantitative yield (entry 9 in Table 2). Additionally, we tested the reaction on a heteroaromatic amide (2-furamide), aliphatic amides (hexanamide and decanamide), and an alicyclic amide (cyclohexanecarboxamide). These substrates yielded the corresponding aldehydes in moderate to good yields (56–78%) with good chemoselectivity (entries 10–13 in Table 2). Next, various *N*,*N*-dimethyl amides were reduced to the corresponding aldehydes in the presence of esters bearing electron-donating and withdrawing groups, as well as an aliphatic ester (Table 3). Irrespective of the nature of the amide substituents, most amides underwent a smooth partial reduction, thereby giving the corresponding aldehydes in quantitative yields. Whereas, aliphatic *N*,*N*-dimethyl amides, gave relatively good yields (78–80%, entries 4, 8, 12 in Table 3).

**Table 2:** Reduction of various *N*,*N*-dimethyl amides in the presence of the ethyl benzoate^a^

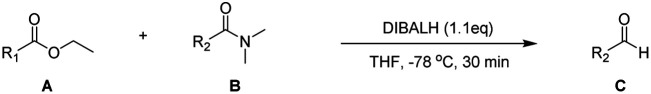					
Entry	Substrate		Product C	Recovery of A^b^ (%)	Yield of C^b^ (%)
	A	B			
1	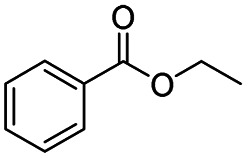	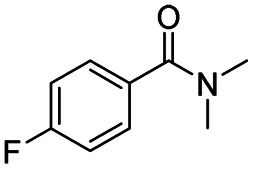	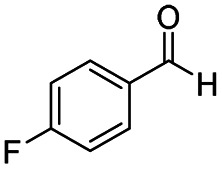	>99	>99
2		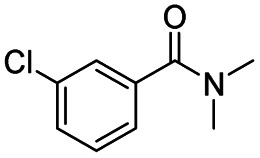	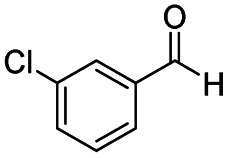	>99	>99
3		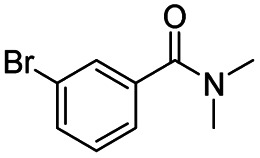	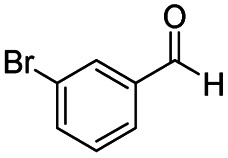	>99	>99
4		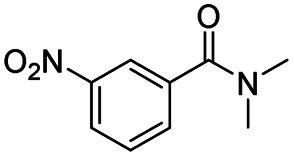	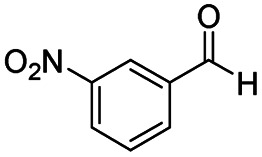	>99	91
5		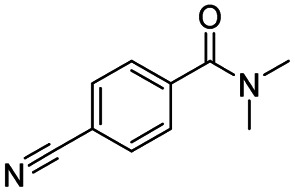	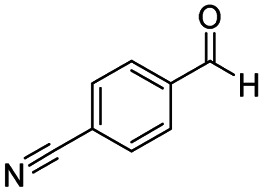	>99	97^f^
6		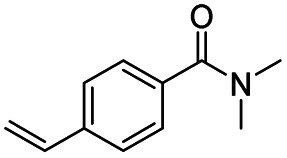	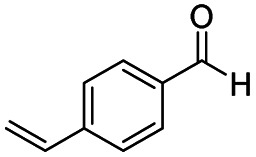	>99	86^f^
7		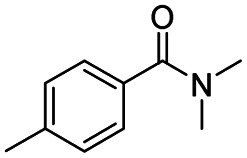	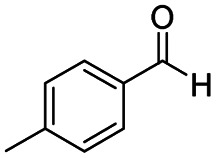	>99	>99^c^
8		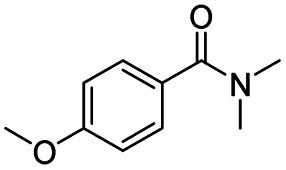	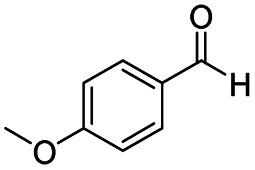	>99	82^d^
9		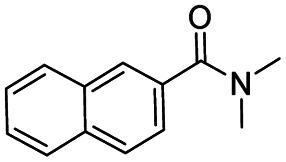	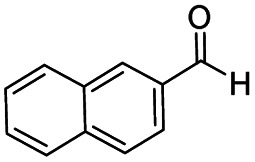	>99	>99
10		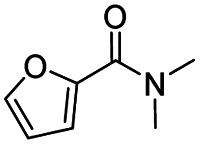	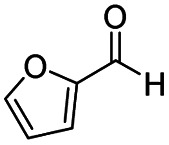	>99	71^e^
11		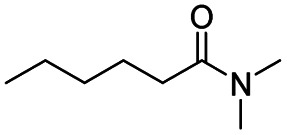	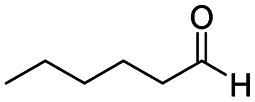	>99	78^e^
12		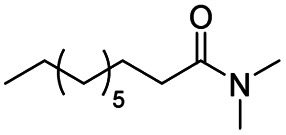	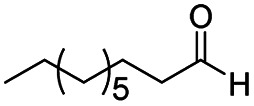	>99	70^f^
13		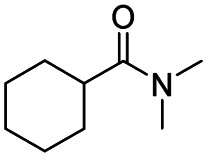	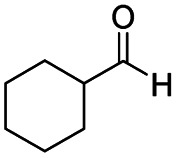	>99	56^g^

a Reaction condition: DIBALH : substrate (1.1 : 1.0), −78 °C, 30 min.

b Yields were determined by GC.

c Reaction time was 1 h.

d Equivalents of substrate : DIBALH = 1 : 1.2.

e Equivalents of substrate : DIBALH = 1 : 1.4.

f Equivalents of substrate : DIBALH = 1 : 1.7.

g Equivalents of substrate : DIBALH = 1 : 1.5.

**Table 3:** Reduction of *N*,*N*-dimethyl amides in the presence of various esters^a^

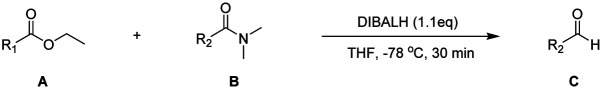					
Entry	Substrate		Product C	Recovery of A^b^ (%)	Yield of C^b^ (%)
	A	B			
1	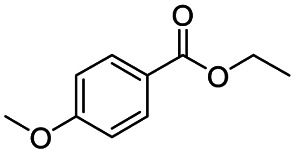	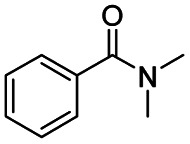	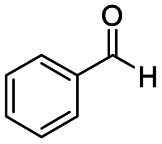	>99	>99
2		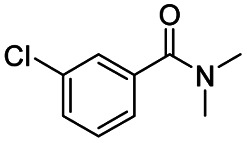	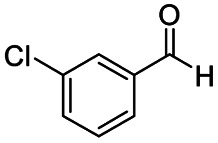	>99	>99
3		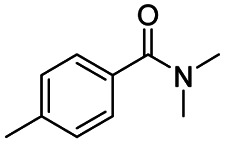	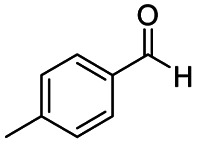	>99	>99^c^
4		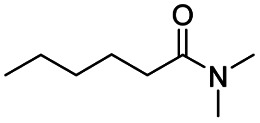	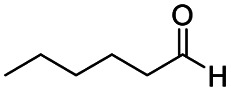	>99	78^d^
5	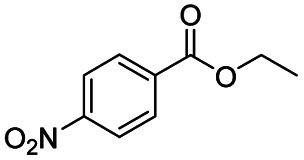	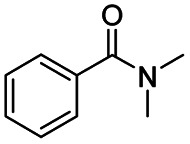	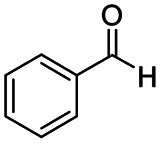	>99	>99
6		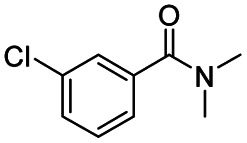	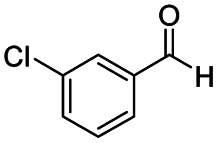	>99	>99
7		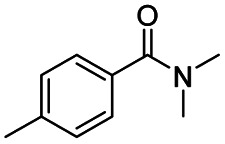	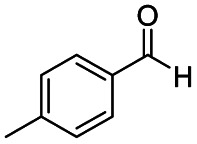	>99	>99^c^
8		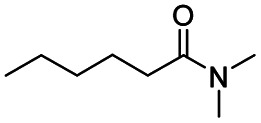	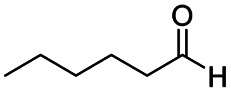	>99	78^d^
9	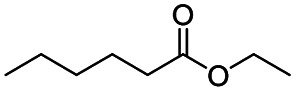	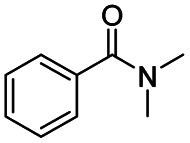	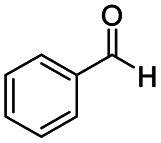	90	>99
10		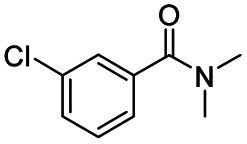	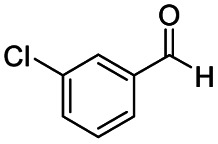	92	>99
11		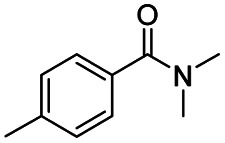	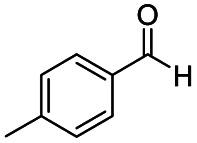	80	>99^e^
12		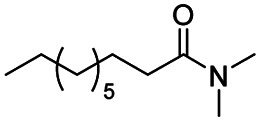	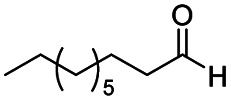	81	80^d^

a Reaction condition: DIBALH : substrate (1.1 : 1.0), −78 °C, 30 min.

b Yields were determined by GC.

c Reaction time was 1 hour.

d Equivalents of substrate : DIBALH = 1 : 1.4.

e Equivalents of substrate : DIBALH = 1 : 1.2.

After achieving the selective reduction of *N*,*N*-dimethyl amides to aldehydes, we turned our attention to the partial reduction of Weinreb and morpholine amides in the presence of various esters. As shown in Table 4, the reduction of both aromatic and aliphatic amides proceeded smoothly to give the corresponding aldehydes in quantitative yields with good chemoselectivity.

**Table 4:** Reduction of various tertiary amides in the presence of esters

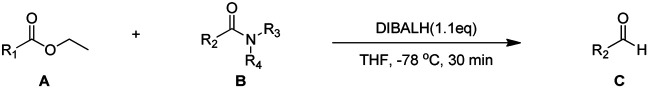					
Entry	Substrate		Product C	Recovery of A^a^ (%)	Yield of C^a^ (%)
	A	B			
1	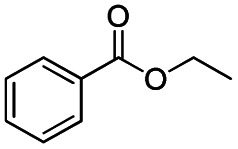	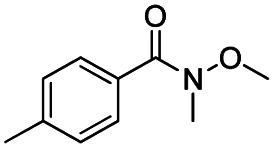	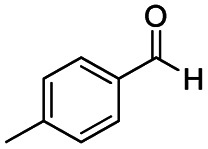	>99	>99
2		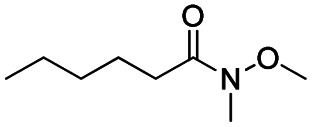	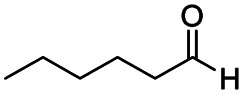	>99	>99
3		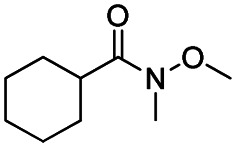	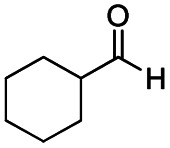	>99	>99
4	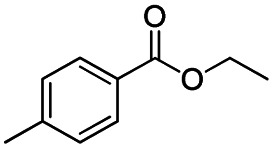	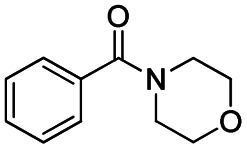	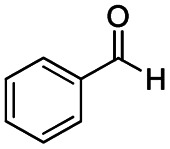	>99	>99
5	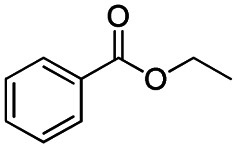	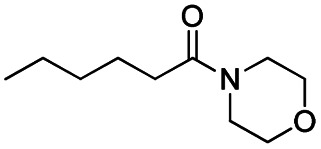	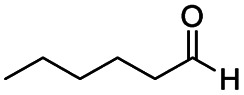	>99	>99
6		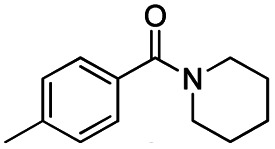	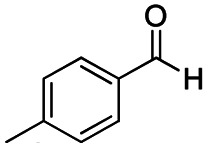	>99	>99

a Yields were determined by GC.

In order to further investigate the chemoselectivity of the present method, we extended it to the multi-functional organic compounds (Table 5). Accordingly, aromatic *N*,*N*-dimethyl amide, morpholine amide, and Weinreb amides containing ester moiety were treated with DIBALH and successive chemoselective reduction was afforded from amide groups leaving esters unreactive in most cases (entries 1–3 in Table 5). Additionally, aliphatic amides with ester groups were converted to aldehydes in good yields (entries 4 and 5 in Table 5).

**Table 5:** Chemoselective reduction of multi-functionalized compounds

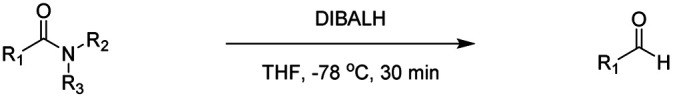				
Entry	Substrate	DIBALH (eq.)	Product	Yield of product^a^ (%)
1	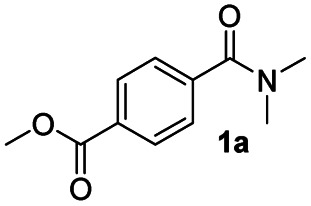	1.1	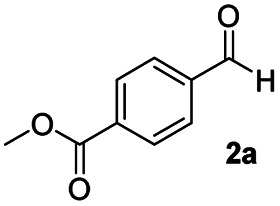	94(93)
2	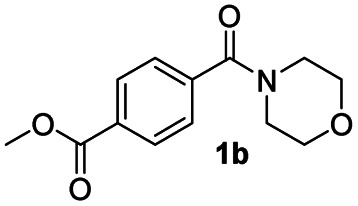	1.2	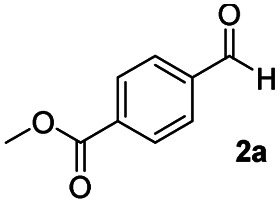	97(90)^b^
3	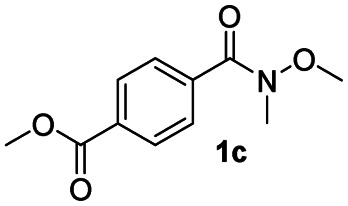	1.2	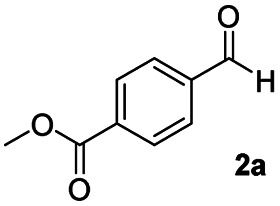	99(84)^b^
4	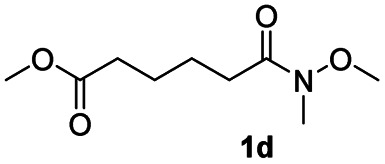	1.1	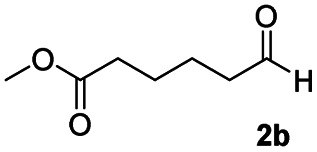	82(75)
5	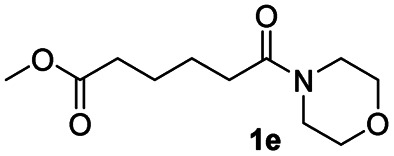	1.1	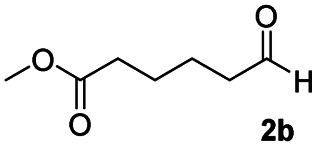	77(73)

a Yields were determined by GC.

b Reaction time is 1 h. The parentheses were isolated yields after silica column chromatography.

## Conclusions

In summary, in this study we have identified an unusual approach for the chemoselective partial reduction of various tertiary amides (*N*,*N*-dimethyl, Weinreb, and morpholine amides) to the corresponding aldehydes in the presence of the relatively more reactive ester groups using commercial DIBALH. Among the tested solvents, THF afforded the best results in terms of chemoselectivity and quantitative conversions. In addition, multi-functional organic compounds also yielded desired aldehydes in the presence of readily reducible ester groups. Therefore, the present method is providing a simple and economically benign protocol for the preparation of aldehydes used in complex syntheses.

## Conflicts of interest

There are no conflicts of interest to declare.

## Supplementary Material

RA-011-D1RA06279D-s001
