# Supplementary material for: DIBALH: from known fundamental to an unusual reaction; chemoselective partial reduction of tertiary amides in the presence of esters
Source: RSC Adv. 2021 Oct 18;11(53):33809–13. doi: 10.1039/d1ra06279d (PMC9042244; doi:10.1039/d1ra06279d)
Supplement: RA-011-D1RA06279D-s001 [file RA-011-D1RA06279D-s001.pdf]

Electronic Supplementary Material (ESI) for RSC Advances.
This journal is © The Royal Society of Chemistry 2021

Supporting Information

DIBALH: From Known Fundamental to an Unusual Reaction; Chemoselective Partial Reduction of Tertiary Amides in the Presence of Esters

Yu Jin Heo, Hyun Tae Kim, Ashok Kumar Jaladi and Duk Keun An*

Department of Chemistry, Kangwon National University

Chuncheon 24341, Republic of Korea

E-mail: dkan@kangwon.ac.kr

Table of Contents

1. Experimental S1

General Information S1

General procedure for intermolecular chemoselective partial reduction of amides to corresponding aldehydes over esters S2

General procedure for Intramolecular chemoselective partial reduction of amides to corresponding aldehydes over esters S2

2. Characterization of the substrate S3-S5

3. Copies of analysis data S6-S30

4. References S31

1. Experimental Section

General Information

All glassware used was dried thoroughly in an oven, assembled hot, and cooled under a stream of dry nitrogen prior to use. All reactions and manipulations of air- and moisture-sensitive materials were carried out using standard techniques for the handling of such materials. All chemicals were commercial products of the highest purity which were further purified before use by using standard methods. HBpin, aldehydes, ketones, alkenes were purchased from Aldrich Chemical Company, Alfa Aesar, and Tokyo Chemical Industry Company (TCI). ^{1}H NMR spectra were measured at 400 MHz with CDCl3 as a solvent at ambient temperature unless otherwise indicated and the chemical shifts were recorded in parts per million downfield from tetramethylsilane (δ = 0 ppm) or based on residual $CDCl_3$ (δ = 7.26 ppm) as the internal standard. ^{13}C NMR spectra were recorded at 101 MHz with $CDCl_3$ as a solvent and referenced to the central line of the solvent (δ = 77.0 ppm). The coupling constants (J) are reported in hertz. Analytical thin-layer chromatography (TLC) was performed on glass precoated with silica gel (Merck, silica gel 60 F254). Column chromatography was carried out using 70-230 mesh silica gel (Merck) at normal pressure. GC analyses were performed on a Younglin Acme 6100M and 6500 GC FID chromatography, using an HP-5 capillary column (30 m). All GC yields were determined with the use of naphthalene as the internal standard and the authentic sample.

General procedure for intermolecular chemoselective partial reduction of amides to corresponding aldehydes over esters (Tables 1-4).

The following experimental procedure for the intermolecular chemoselective partial reduction of ethyl benzoate and *N,N*-dimethylbenzamide is representative. A dry and argon-flushed flask, equipped with a magnetic stirring bar, *N,N*-dimethylbenzamide (74.6 mg, 0.5 mmol) and a septum, was charged with ethyl benzoate (0.07 mL, 0.5 mmol) and 5 mL THF. After cooling to -78 ℃, DIBALH (0.55 mL, 1.0 M in hexane soln., 0.55 mmol) was slowly added and stirred for 30 min at the same temperature. After completion of reaction (GC), it was quenched by the addition of aqueous 1 *N* HCl (5 mL) at room temperature and extracted with diethyl ether (2 x 10 mL). The combined organic layers were dried over $MgSO_4$. GC analysis showed a 99% recovery yield of ethyl benzoate and 99% yield of benzaldehyde. All products in Tables 1-4 were confirmed through comparison with GC data of authentic sample.

General procedure for intramolecular chemoselective partial reduction of amides to corresponding aldehydes over esters (Table 5).

The following experimental procedure for the intramolecular chemoselective partial reduction of methyl 4-(dimethylcarbamoyl)benzoate is representative. A dry and argon-flushed flask, equipped with a magnetic stirring bar, methyl 4-(dimethylcarbamoyl)benzoate (207 mg, 1.0 mmol) and a septum, was charged 10 mL THF. After cooling to -78 ℃, DIBALH (1.1 mL, 1.0 M in hexane soln., 1.1 mmol) was slowly added and stirred for 30 min at the same temperature. After completion of reaction (GC), it was quenched by the addition of aqueous 1 *N* HCl (5 mL) at room temperature and extracted with diethyl ether (2 x 10 mL). The combined organic layers were dried over $MgSO_4$. GC analysis showed a 94% yield of methyl 4-formylbenzoate. After filtered and concentrated under reduced pressure. Residue was purified by column chromatography on silica gel yielded methyl 4-formylbenzoate (152 mg, 93%). All products in Tables 5 were confirmed through comparison with GC data of authentic sample or spectral data of the reported literature.

2. Characterization of the substrate.

N,N-dimethyl-4-cyanobenzamide (Entry 5 in Table 2)

The 50 mL two necked round bottom with magnetic bar and septum was charged with dimethylamine hydrochloride (5.910 g, 72.47 mmol, 4.0 eq) and methylene chloride (MC, 21.3 mL). To this, NEt_3 (10.1 ml, 72.47 mmol, 4.0 eq) and 4-cyanobenzoyl chloride (3.0 g, 18.12 mmol, 1.0 eq) were added at 0 ℃, stirred at room temperature (25 ℃). After completion of reaction (TLC), crude mixture was extracted with MC, washed with 1 N HCl (aq), and brine solution. The organic part was dried over $MgSO_4$, filtered and dried under reduced pressure. Crude compound was purified by silica gel chromatography in 80% yield as white powder. ^{1}H NMR (400 MHz, $CDCl_3$) δ 7.70 (d, *J* = 7.6 Hz, 2H), 7.50 (d, *J* = 7.6 Hz, 2H), 3.11 (s, 3H), 2.94 (s, 3H), ^{13}C NMR (101 MHz, $CDCl_3$) δ 169.61, 140.76, 132.43, 127.84, 118.25, 113.44, 39.43, 35.44. NMR data was in accordance with reported literature[S1]

N,N-dimethyl-4-vinylbenzamide (Entry 6 in Table 2)

The 50 mL two necked round bottom with magnetic bar and septum was charged with 4-vinylbenzoic acid (3.0 g, 20.25 mmol, 1.0 eq) and 1,1'-carbonyldiimidazole (3.61 g, 22.27 mmol, 1.1 eq) and methylene chloride (MC, 78.9 mL), stirred of 1 h. To this, NEt_3 (11.23 ml, 81.0 mmol, 4.0 eq) and dimethylamine hydrochloride (3.30 g, 40.5 mmol, 2.0 eq) were added at 25 ºC, stirred for overnight. After completion of reaction (monitored by TLC), crude mixture was extracted with MC, washed with water and brine solution. The organic part was dried over $MgSO_4$, filtered and dried under reduced pressure. Crude compound was purified by silica gel chromatography in 94% yield as white powder. 1H NMR (400 MHz, $CDCl_3$) δ 7.39 (q, J = 8.3 Hz, 4H), 6.71 (dd, J = 17.6, 10.9 Hz, 1H), 5.78 (d, J = 17.6 Hz, 2H), 5.30 (d, J = 10.9 Hz, 2H), 3.03 (d, J = 45.7 Hz, 6H). ^{13}C NMR (101 MHz, $CDCl_3$) δ 171.47, 138.84, 136.20, 135.57, 127.56, 126.17, 115.24. NMR data was in accordance with reported literature[S2]

Methyl 4-(dimethylcarbamoyl)benzoate (1a):

This compound was prepared from methyl 4-(chlorocarbonyl)benzoate and dimethylamine in 53% yield: ^{1}H-NMR (400 MHz, $CDCl_3$) δ: 8.01 (d, *J* = 8.0 Hz, 2H), 7.41 (d, *J* = 8.0 Hz, 2H), 3.86 (s, 3H), 3.05 (s, 3H), 2.88 (s, 3H). ^{13}C NMR (101 MHz, $CDCl_3$) δ: 170.59, 166.44, 140.72, 130.99, 129.75, 127.05, 52.36, 39.43, 35.31. NMR data was in accordance with reported literature[S3]

Methyl 4-(morpholine-4-carbonyl)benzoate (1b):

This compound was prepared from methyl 4-(chlorocarbonyl)benzoate and morpholine in 61% yield: ^{1}H-NMR (400 MHz, $CDCl_3$) δ: 8.08 (d, *J* = 8.1 Hz, 2H), 7.46 (d, *J* = 8.1 Hz, 2H), 3.92 (s, 3H),

3.84 – 3.32 (m, 8H). ^{13}C NMR (101 MHz, $CDCl_3$) δ: 169.45, 166.35, 139.66, 131.42, 129.99, 127.15, 66.91, 52.47, 48.15, 42.53. NMR data was in accordance with reported literature[S4]

Methyl 4-(N-methoxy-N-methyl carbamoyl)benzoate (**1c**):

A solution of K_2CO_3 in THF was cooled to 0 °C and treated with *N,O*-dimethylhydroxyamine hydrochloride in portions. To the reaction was added methyl 4-(chlorocarbonyl)benzoate dropwise and the resultant mixture was warmed to room temperature for 6 h. The reaction was diluted with water and extracted with EtOAc and the organic layer was dried ($MgSO_4$). The solvent was removed and crude product was purified by column chromatography using 1:1 hexanes/EtOAc to give Methyl 4-(N-methoxy-N-methyl carbamoyl)benzoate (**1c**) in 42% yield: ^{1}H-NMR (400 MHz, $CDCl_3$) δ: 8.06 (d, J = 8.6 Hz, 2H), 7.71 (d, J = 8.5 Hz, 2H), 3.93 (s, 3H), 3.52 (s, 3H), 3.36 (s, 3H). ^{13}C NMR (101 MHz, $CDCl_3$) δ: 169.11, 166.52, 138.41, 131.85, 129.35, 128.17, 61.30, 52.43, 33.52. NMR data was in accordance with reported literature[S5]

Methyl 6-(methoxy(methyl)amino)-6-oxohexanoate (**1d**):

This compound was prepared from methyl 6-(dimethylamino)-6-oxohexanoate and morpholine in 35% yield: ^{1}H-NMR (400 MHz, $CDCl_3$) δ: 3.67 (d, J = 5.2 Hz, 6H), 3.17 (s, 3H), 2.44 (t, J = 6.0 Hz, 3H), 2.35 (t, J = 7.1 Hz, 2H), 1.68 (td, J = 5.9, 4.9, 3.4 Hz, 6H). ^{13}C NMR (101 MHz, $CDCl_3$) δ: 173.89, 61.21, 51.48, 33.82, 32.09, 31.40, 24.62, 24.30, 24.00. HRMS (DIP-EI): *m/z* [M] calculated for $C_9H_{17}NO_4$: 203.1158; found: 203.1157.

Methyl 6-morpholine-6-oxohexanoate (**1e**):

This compound was prepared from methyl 6-(dimethylamino)-6-oxohexanoate and *N,O*-dimethylhydroxyamine hydrochloride in 42% yield: ^{1}H-NMR (400 MHz, $CDCl_3$) δ: 3.62 (d, J = 3.9 Hz, 7H), 3.59 – 3.52 (m, 2H), 3.44 – 3.35 (m, 2H), 2.30 (td, J = 7.3, 5.0 Hz, 4H), 1.67 – 1.59 (m, 4H). ^{13}C NMR (101 MHz, $CDCl_3$) δ: 173.91, 171.25, 66.97, 66.69, 51.60, 46.00, 41.93, 33.84, 32.71, 24.62. NMR data was in accordance with reported literature[S6]

Methyl 4-formylbenzoate (**2a**):

A 25 mL two-necked RBF with stirring bar was charged with methyl 4-(dimethylcarbamoyl)benzoate (**1a**, 1.0 mmol) and 10 mL THF. After cooling to -78 °C, DIBALH (1.1 mL, 1.0 M in hexane soln., 1.1 mmol) was slowly added and stirred for 30 min at the same temperature. After completion of reaction, it was quenched by the addition of aqueous 1 *N* HCl (5 mL) at room temperature and extracted with diethyl ether (2 x 10 mL). The combined organic layers were dried over $MgSO_4$ and crude product was purified by

column chromatography to afford Methyl 4-formylbenzoate (**2a**) in 93% yield: ^{1}H-NMR (400 MHz, $CDCl_3$) δ: 10.06 (s, 1H), 8.16 (d, *J* = 8.1 Hz, 2H), 7.91 (d, *J* = 8.6 Hz, 2H), 3.92 (s, 3H). ^{13}C NMR (101 MHz, $CDCl_3$) δ: 191.74, 166.11, 139.19, 135.13, 130.25, 129.58, 52.66. NMR data was in accordance with reported literature[S7]

Methyl 6-oxohexanoate (**2b**) **from 6-(methoxy(methyl)amino)-6-oxohexanoate (1d)**:

A 25 mL two-necked RBF with stirring bar was charged with methyl 6-(methoxy(methyl)amino)-6-oxohexanoate (**1d**, 1.0 mmol) was charged 10 mL THF. After cooling -78 °C, DIBALH (1.1 mL, 1.0 M in hexane soln., 1.1 mmol) was slowly added and stirred for 30 min at the same temperature. After completion of reaction, it was quenched by the addition of aqueous 1 *N* HCl (5 mL) at room temperature and extracted with diethyl ether (2 x 10 mL). The combined organic layers were dried over $MgSO_4$ and crude product was purified by column chromatography to afford methyl 6-oxohexanoate (**2b**) in 75% yield: ^{1}H-NMR (400 MHz, $CDCl_3$) δ: 9.75 (s, 1H), 3.65 (s, 3H), 2.45 (ddq, *J* = 5.8, 4.4, 1.5 Hz, 2H), 2.32 (tq, *J* = 4.5, 1.3 Hz, 2H), 1.67 – 1.63 (m, 4H). ^{13}C NMR (101 MHz, $CDCl_3$) δ: 202.15, 173.78, 51.67, 43.58, 33.79, 24.42, 21.57. NMR data was in accordance with reported literature[S8]

3. Copies of analysis data.

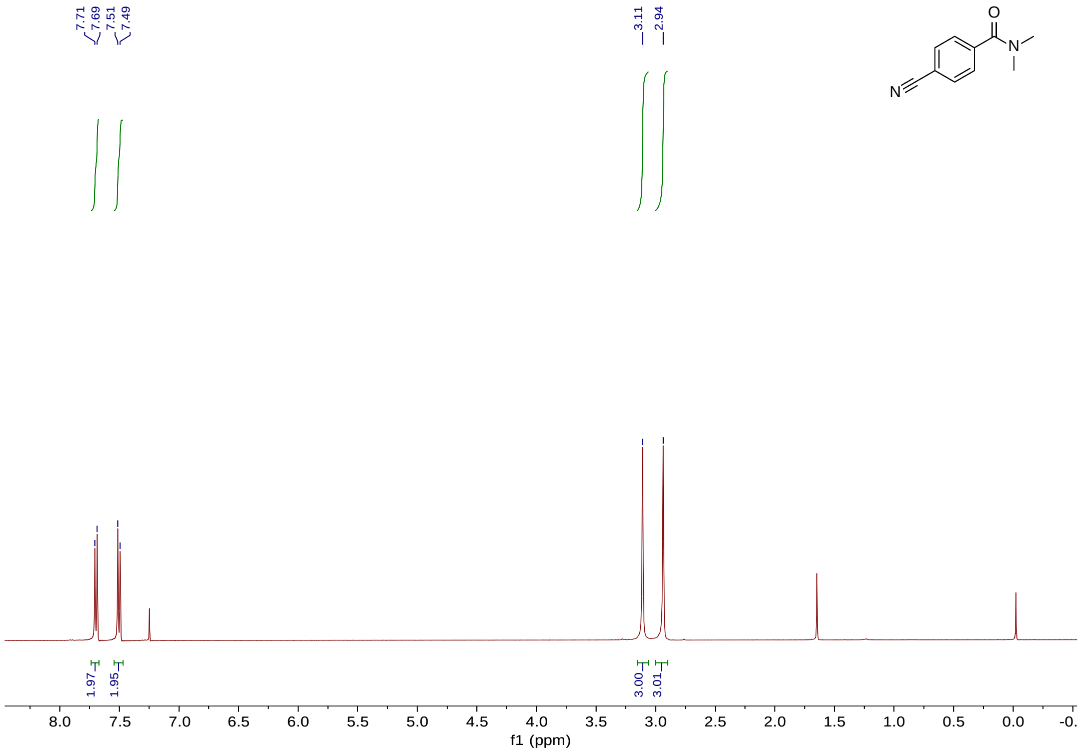

Figure S1: ^{1}H NMR of N,N-dimethyl-4-cyanobenzamide (Entry 5 in Table 2)

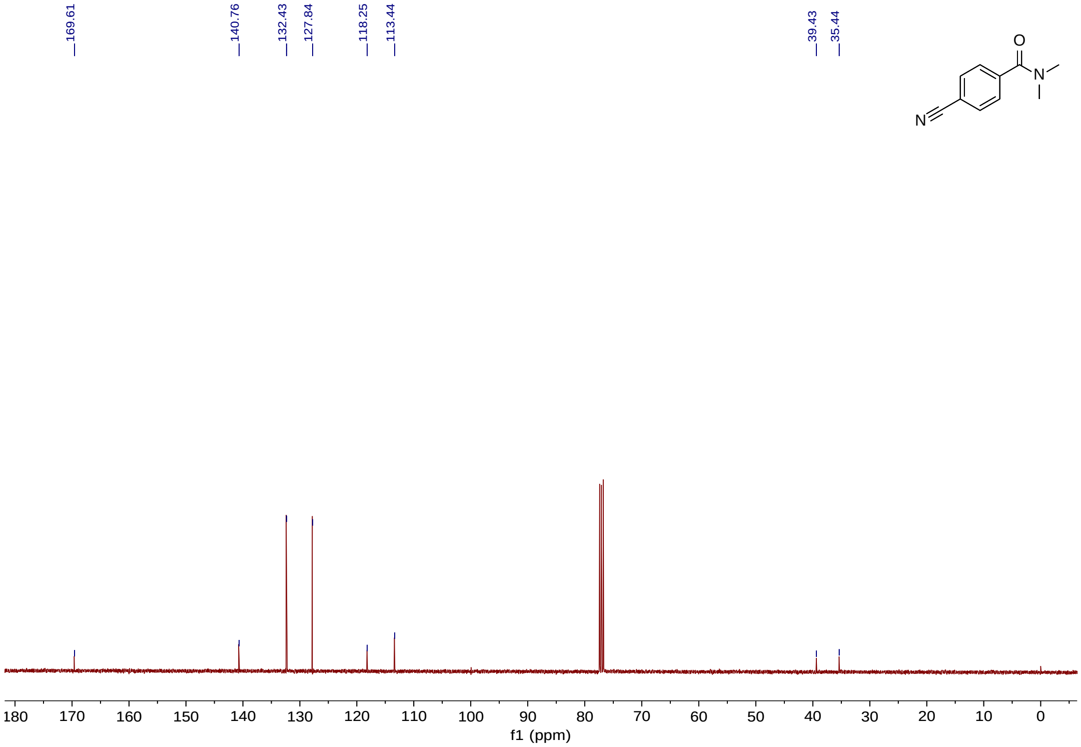

Figure S2: ^{13}C NMR of N,N-dimethyl-4-cyanobenzamide (Entry 5 in Table 2)

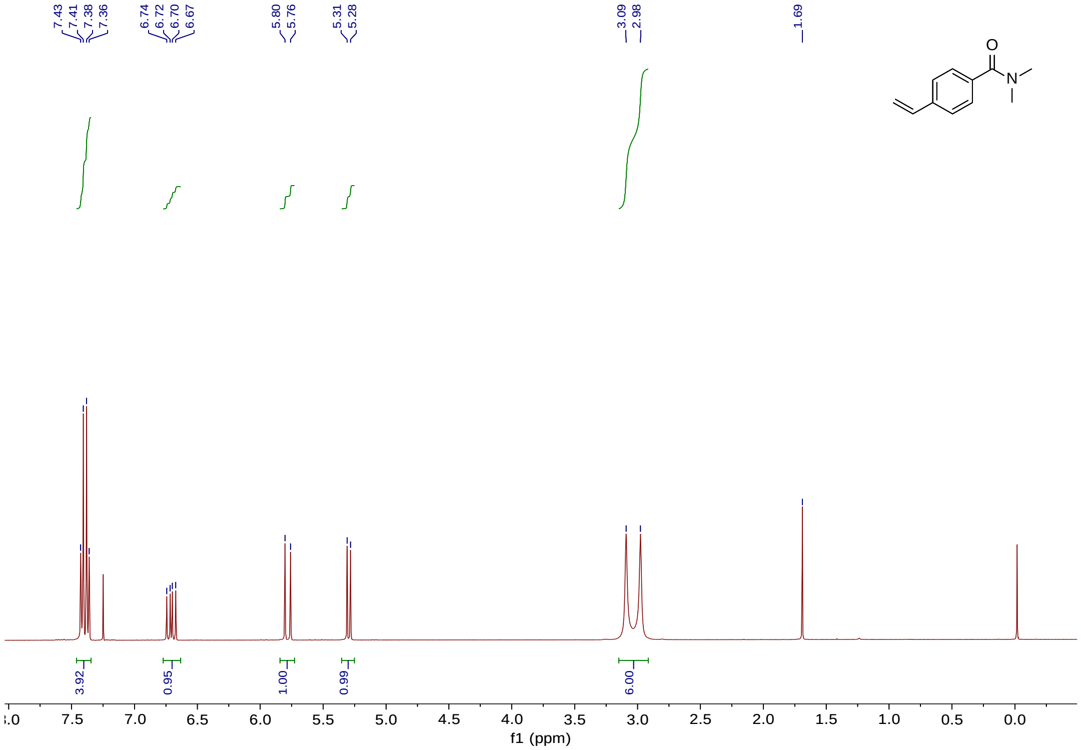

Figure S3: ^{1}H NMR of N,N-dimethyl-4-vinylbenzamide (Entry 6 in Table 2)

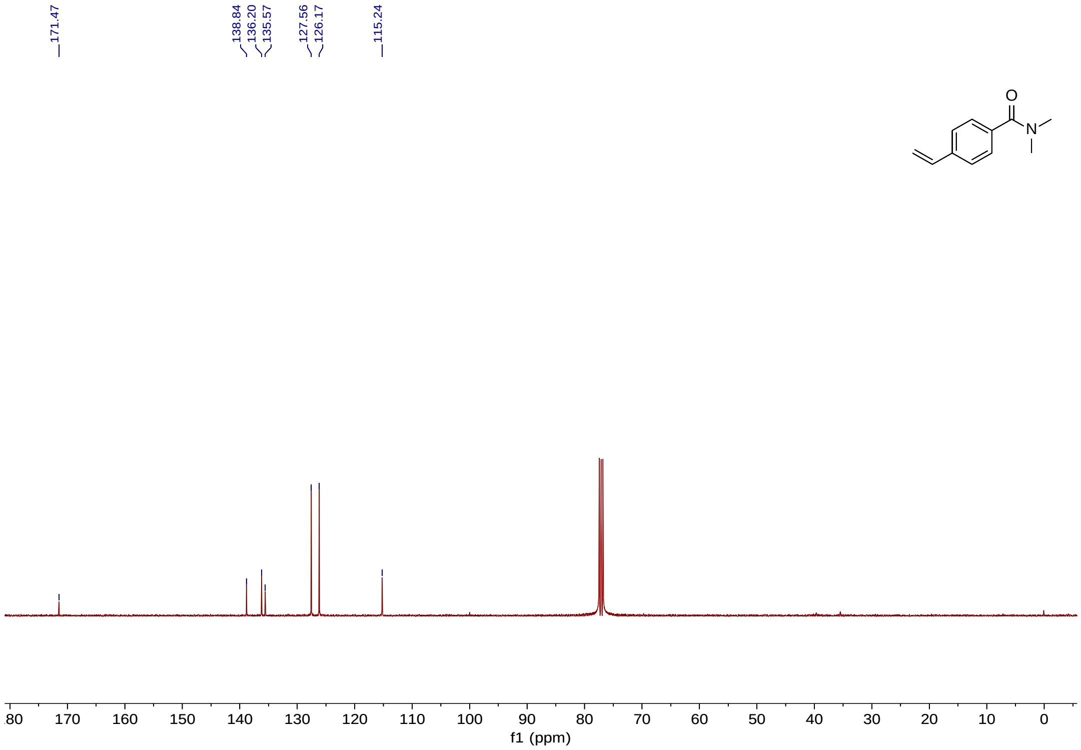

Figure S4: ^{13}C NMR of N,N-dimethyl-4-vinylbenzamide (Entry 6 in Table 2)

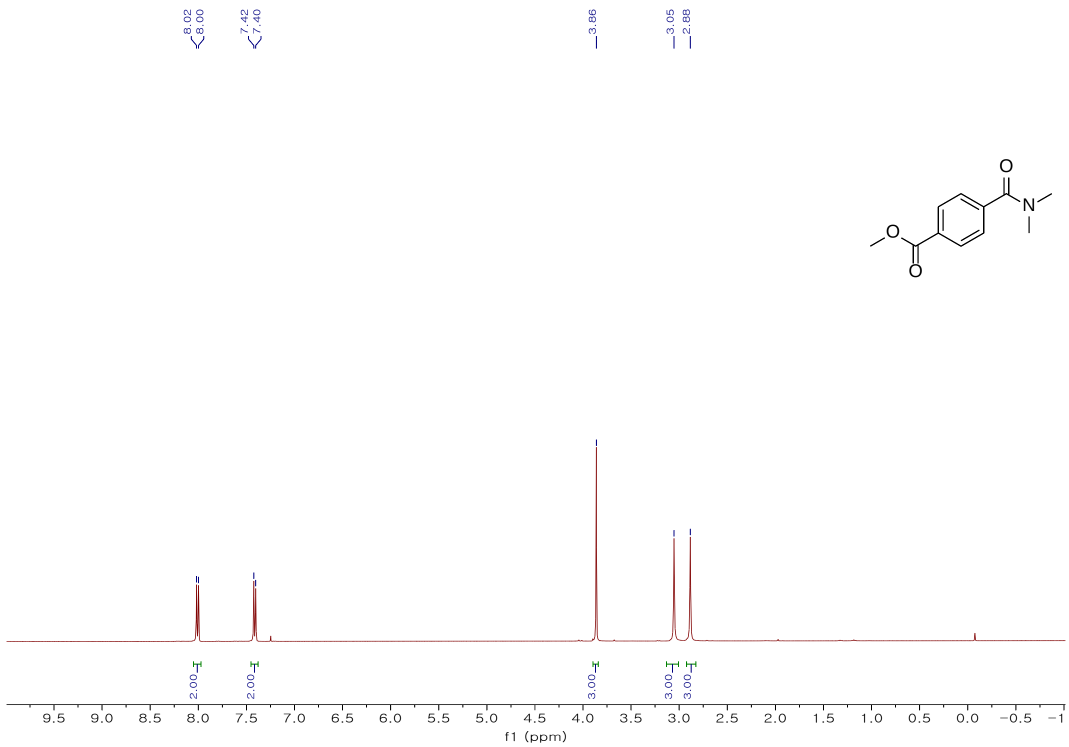

Figure S5: ^{1}H NMR of Methyl 4-(dimethylcarbamoyl)benzoate (**1a**)

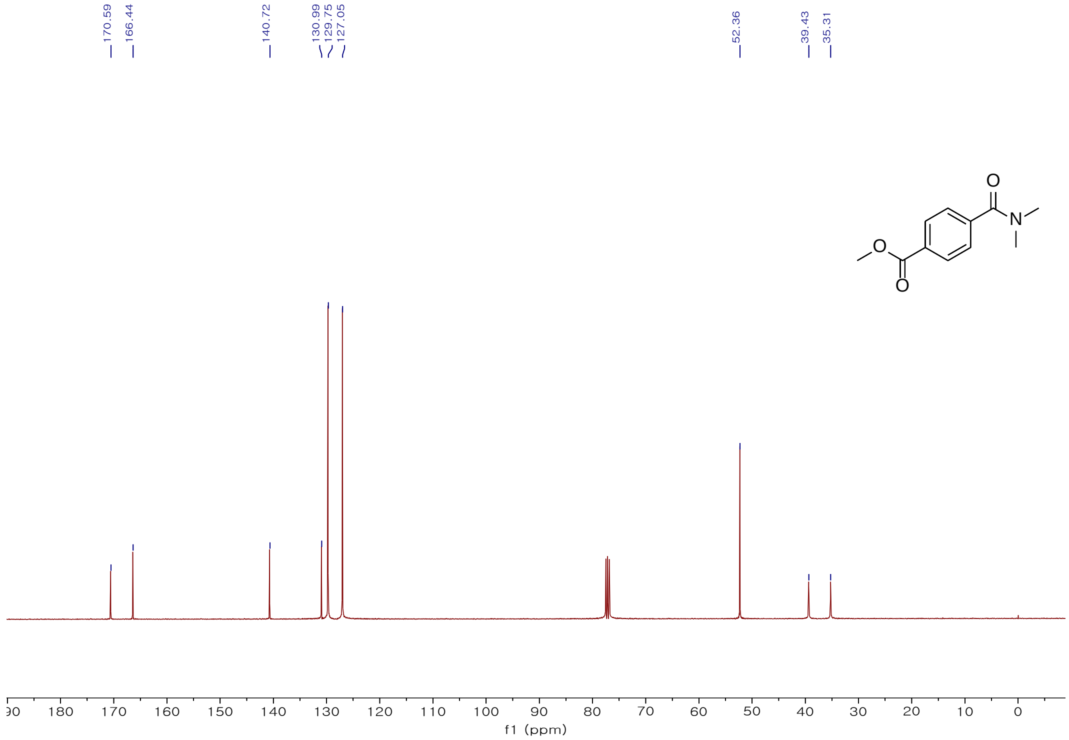

Figure S6: ^{13}C NMR of Methyl 4-(dimethylcarbamoyl)benzoate (**1a**)

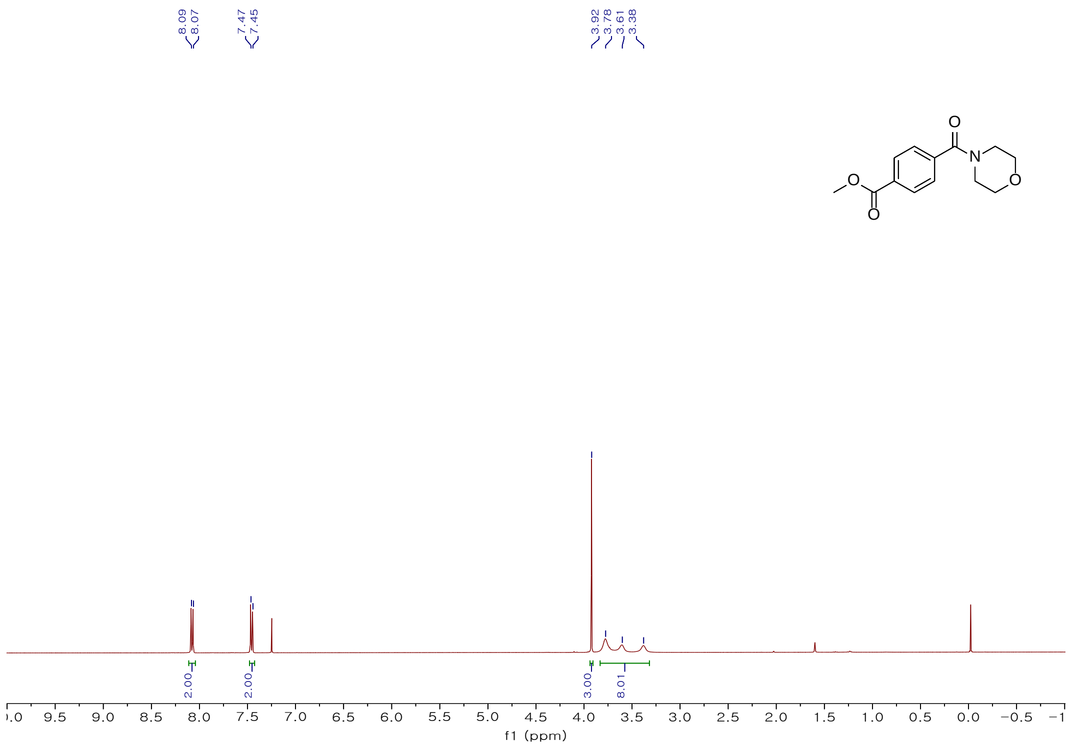

Figure S7: ^{1}H NMR of Methyl 4-(morpholine-4-carbonyl)benzoate (**1b**)

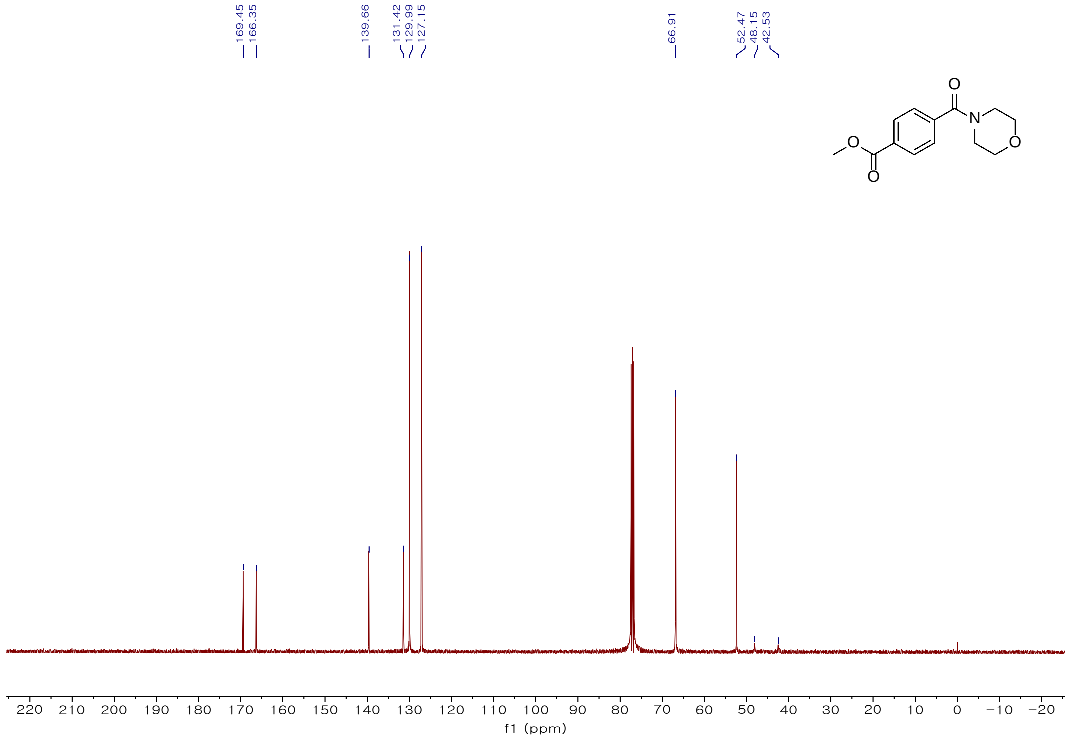

Figure S8: ^{13}C NMR of Methyl 4-(morpholine-4-carbonyl)benzoate (**1b**)

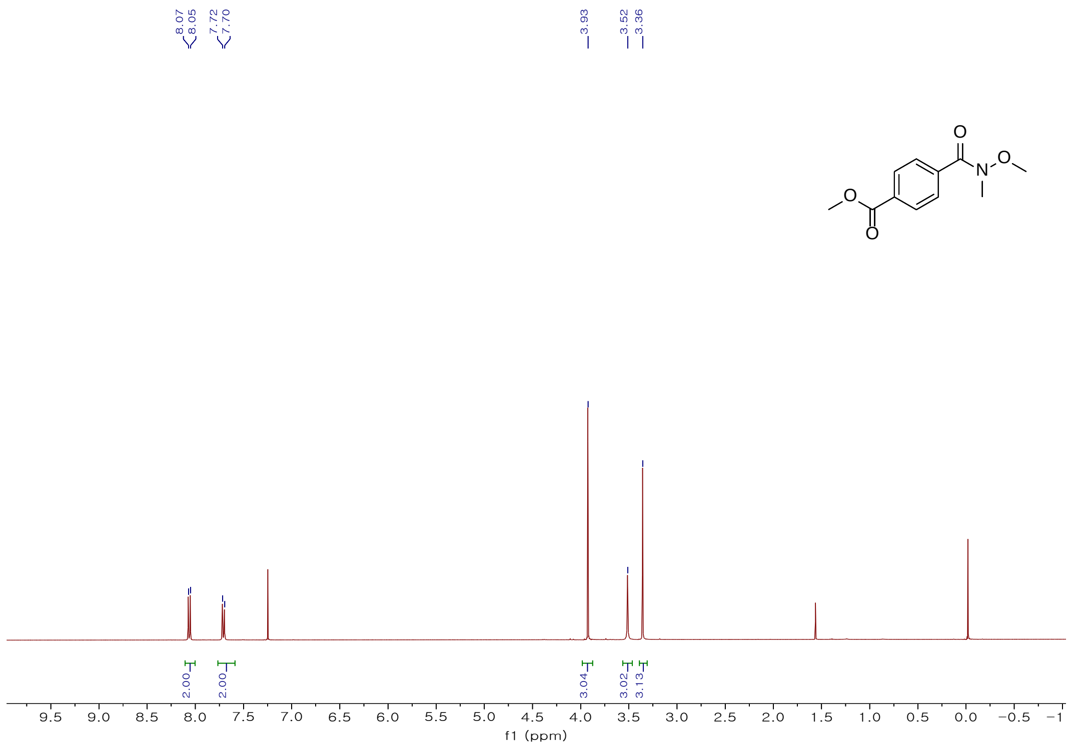

Figure S9: ^{1}H NMR of Methyl 4-(N-methoxy-N-methyl carbamoyl)benzoate (**1c**)

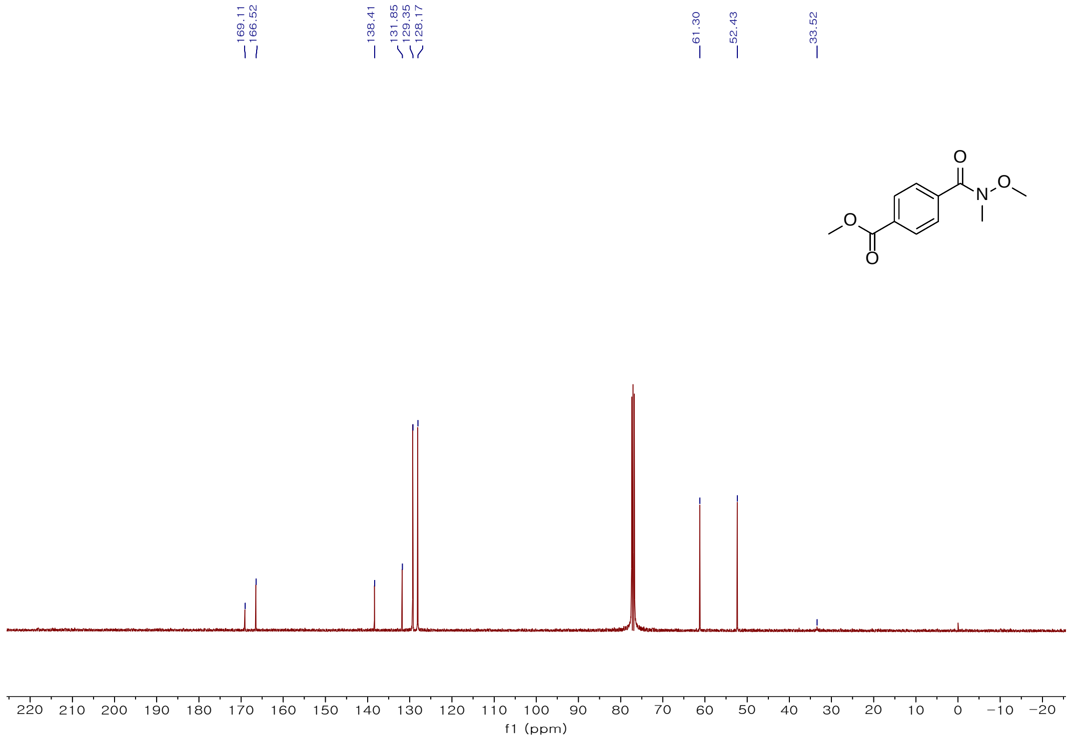

Figure S10: ^{13}C NMR of Methyl 4-(N-methoxy-N-methyl carbamoyl)benzoate (**1c**)

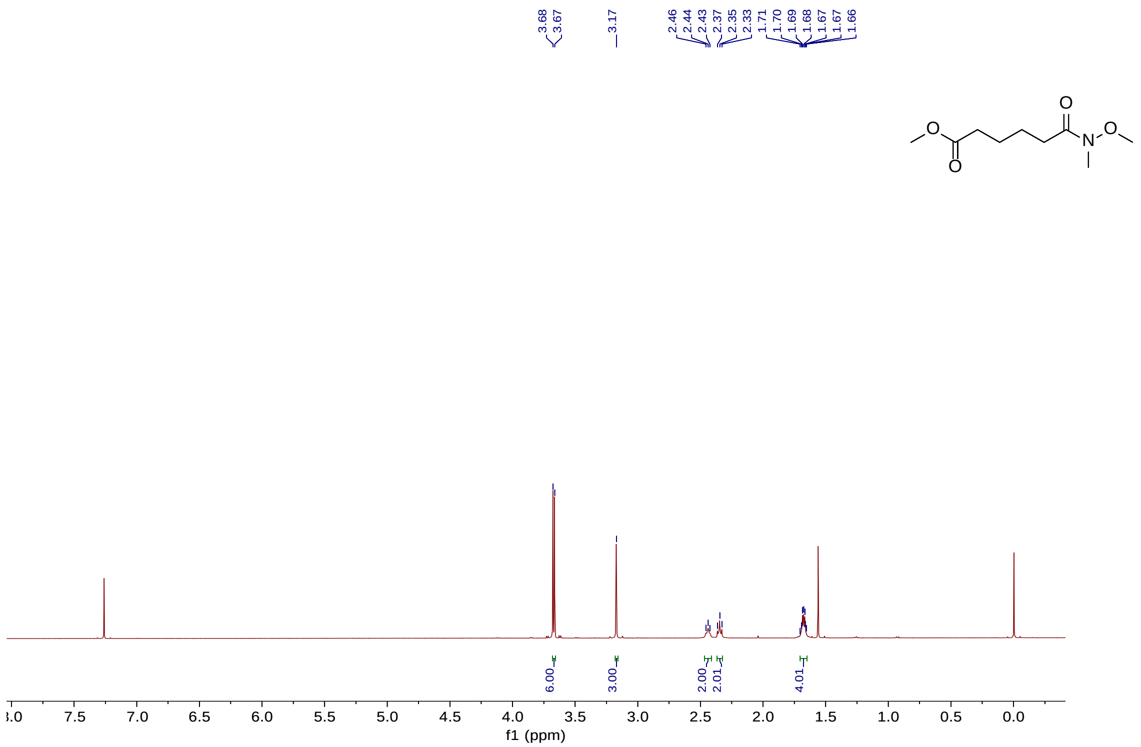

Figure S11: ^{1}H NMR of Methyl 6-(methoxy(methyl)amino)-6-oxohexanoate (**1d**)

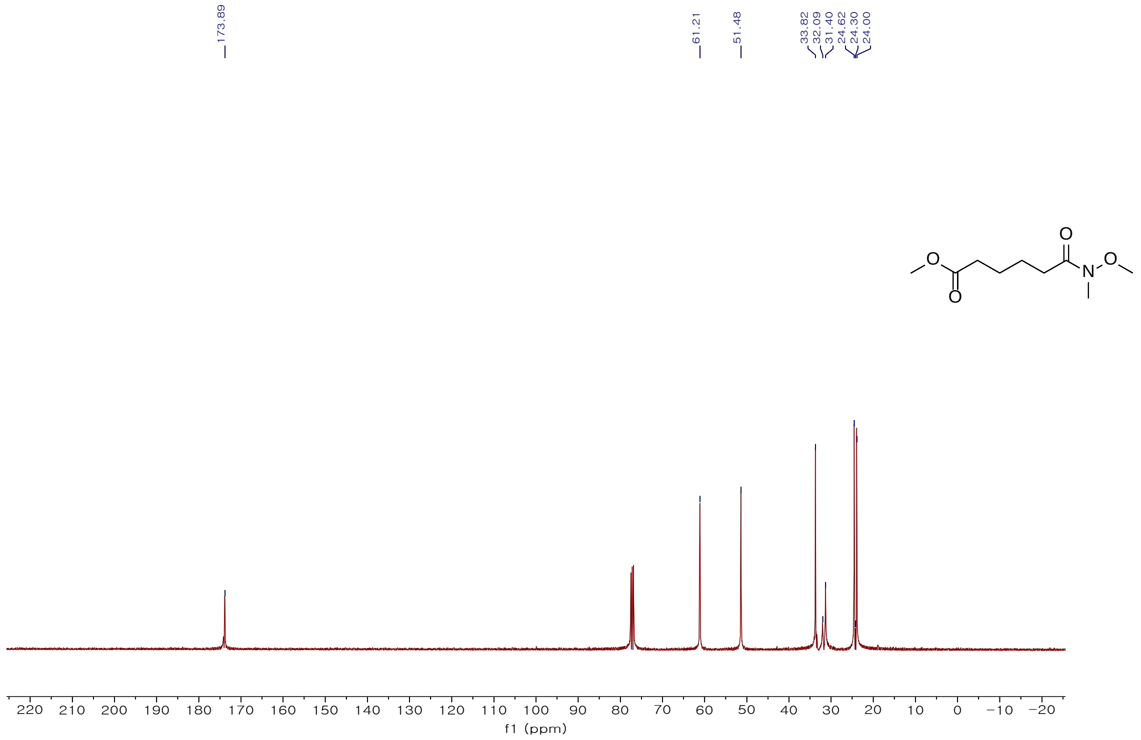

Figure S12: ^{13}C NMR of Methyl 6-(methoxy(methyl)amino)-6-oxohexanoate (**1d**)

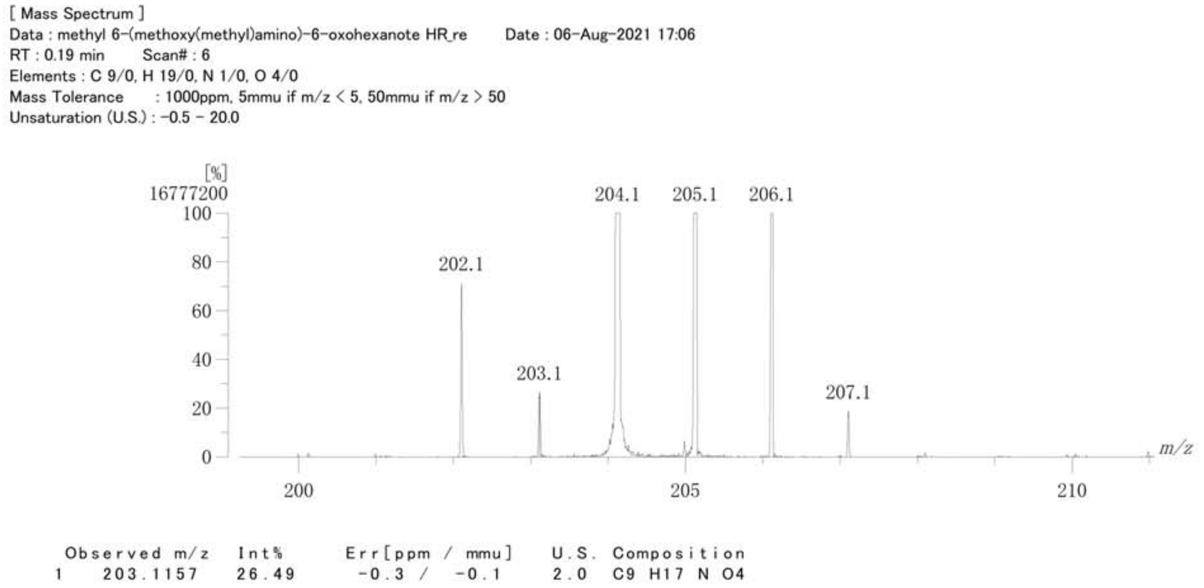

Figure S13: HRMS of Methyl 6-(methoxy(methyl)amino)-6-oxohexanoate (**1d**)

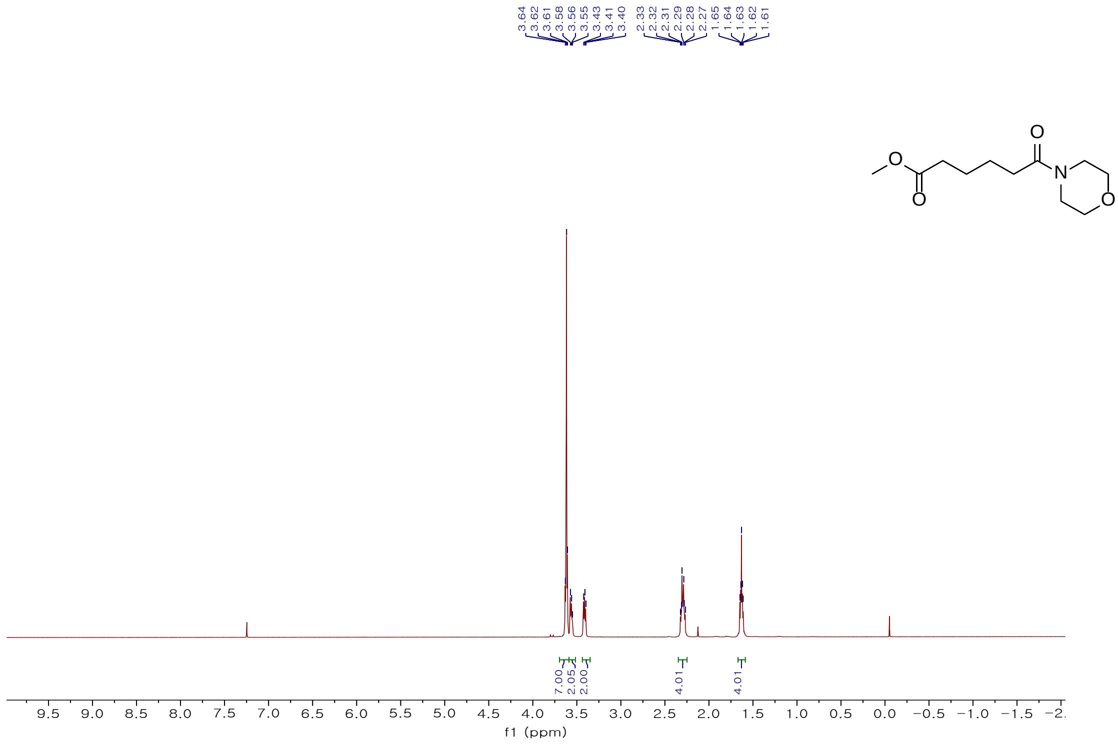

Figure S14: ^{1}H NMR of Methyl 6-morpholine-6-oxohexanoate (**1e**)

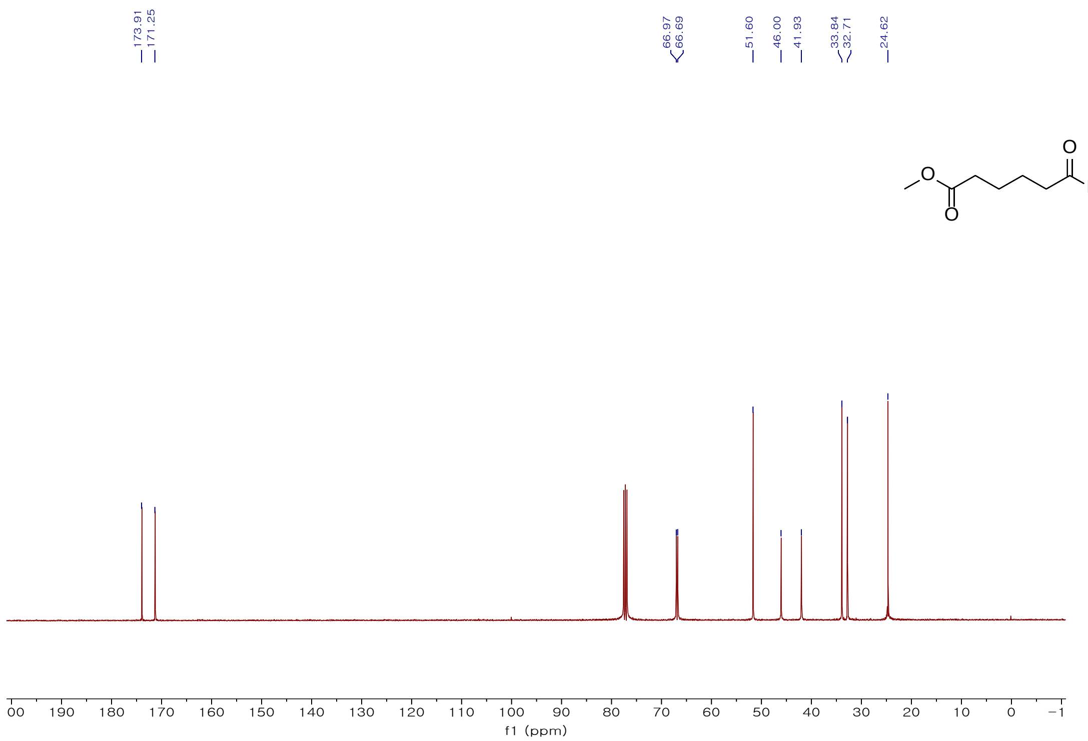

Figure S15: ^{13}C NMR of Methyl 6-morpholine-6-oxohexanoate (**1e**)

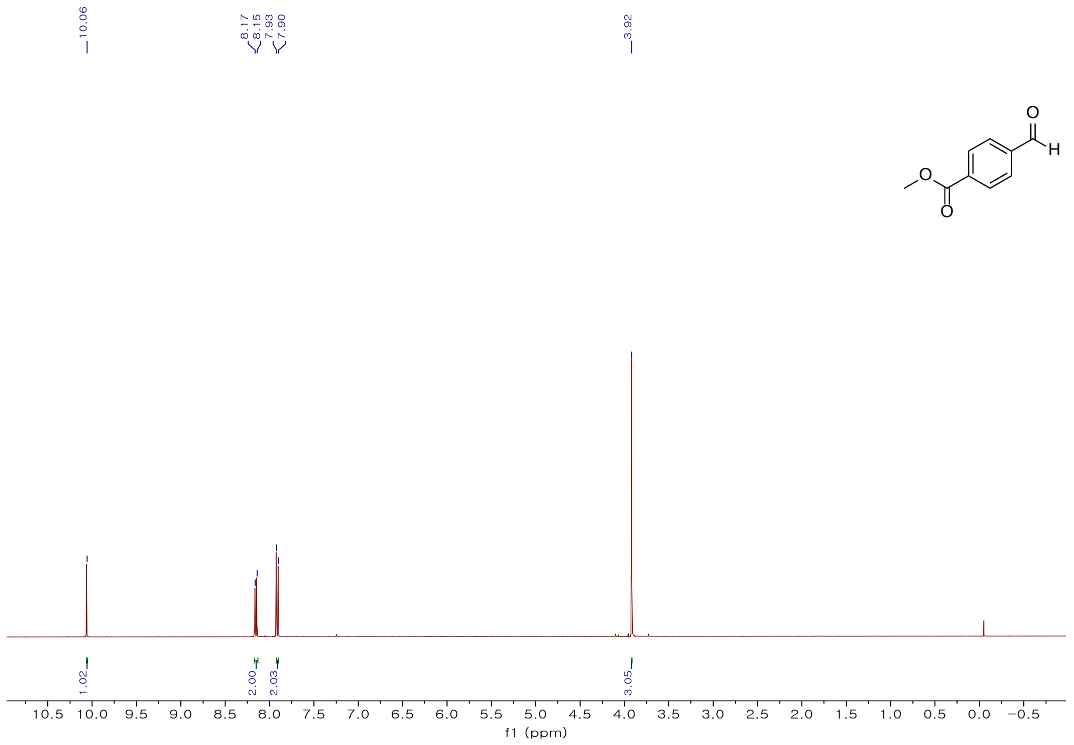

Figure S16: ^{1}H NMR of Methyl 4-formylbenzoate (**2a**)

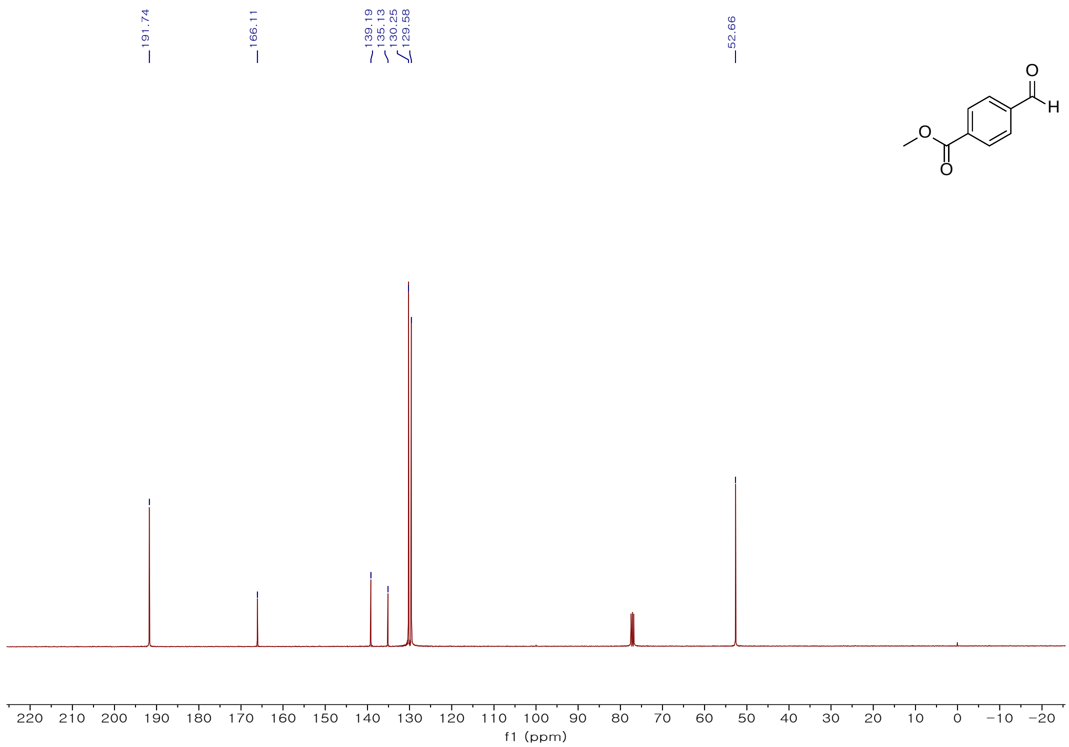

Figure S17: ^{13}C NMR of Methyl 4-formylbenzoate (**2a**)

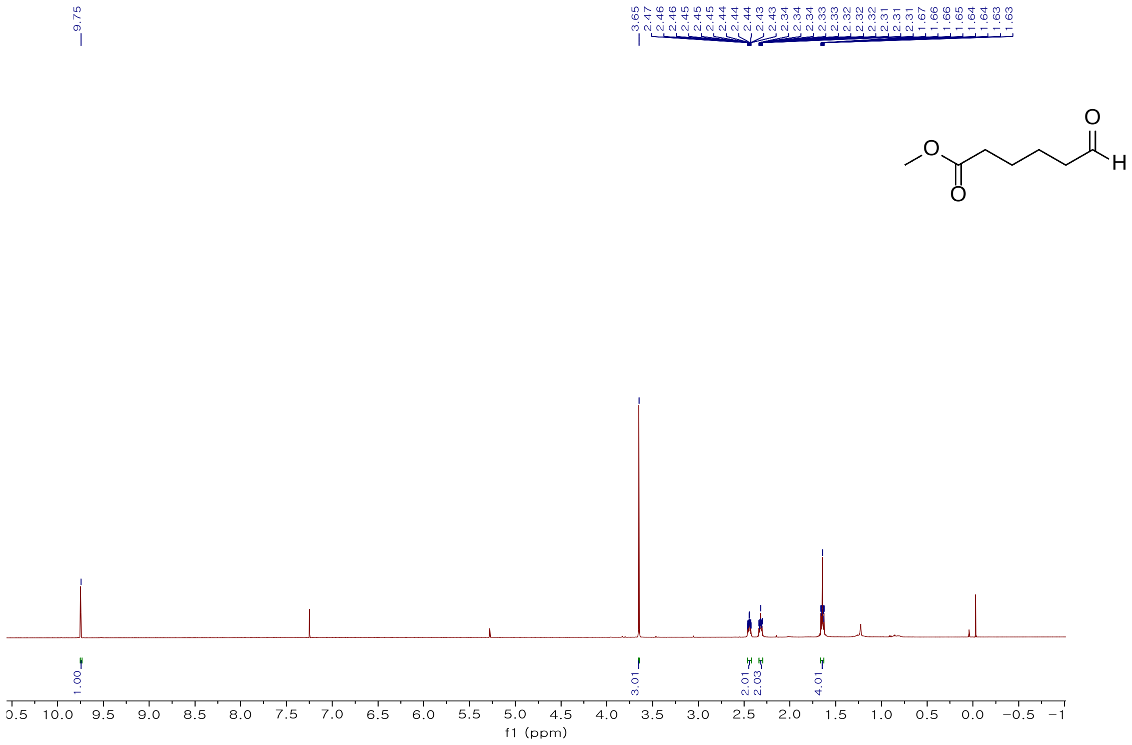

Figure S18: ^{1}H NMR of Methyl 6-oxohexanoate (**2b**)

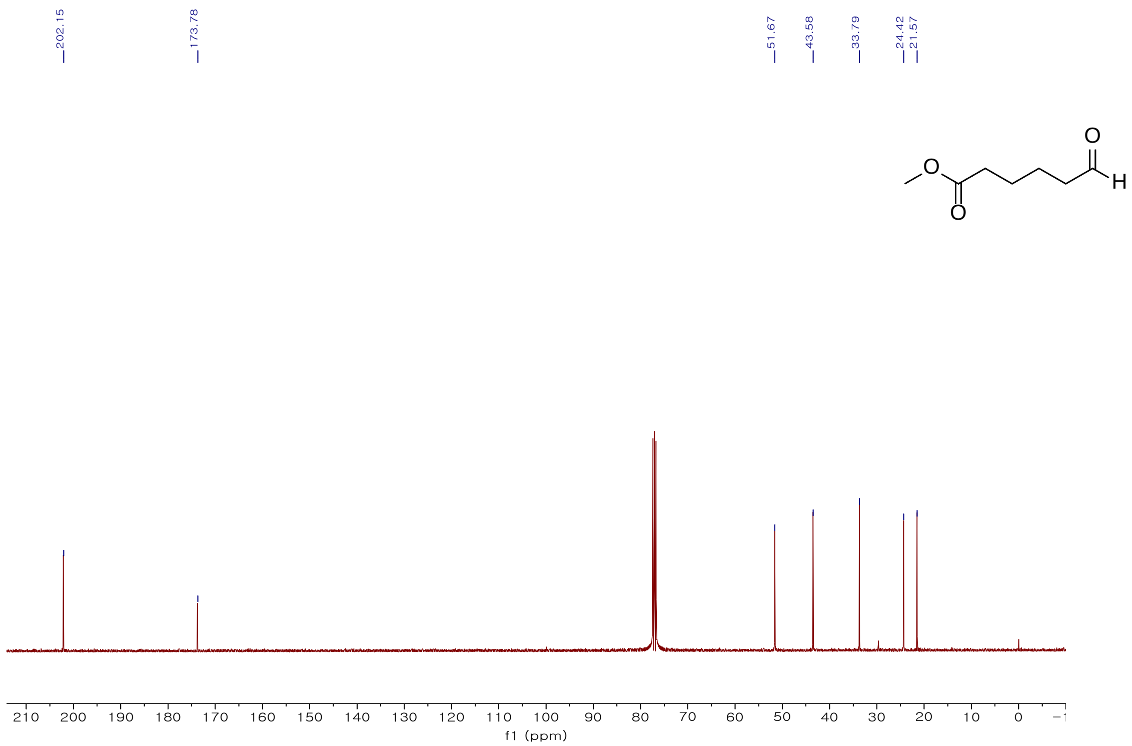

Figure S19: ^{13}C NMR of Methyl 6-oxohexanoate (**2b**)

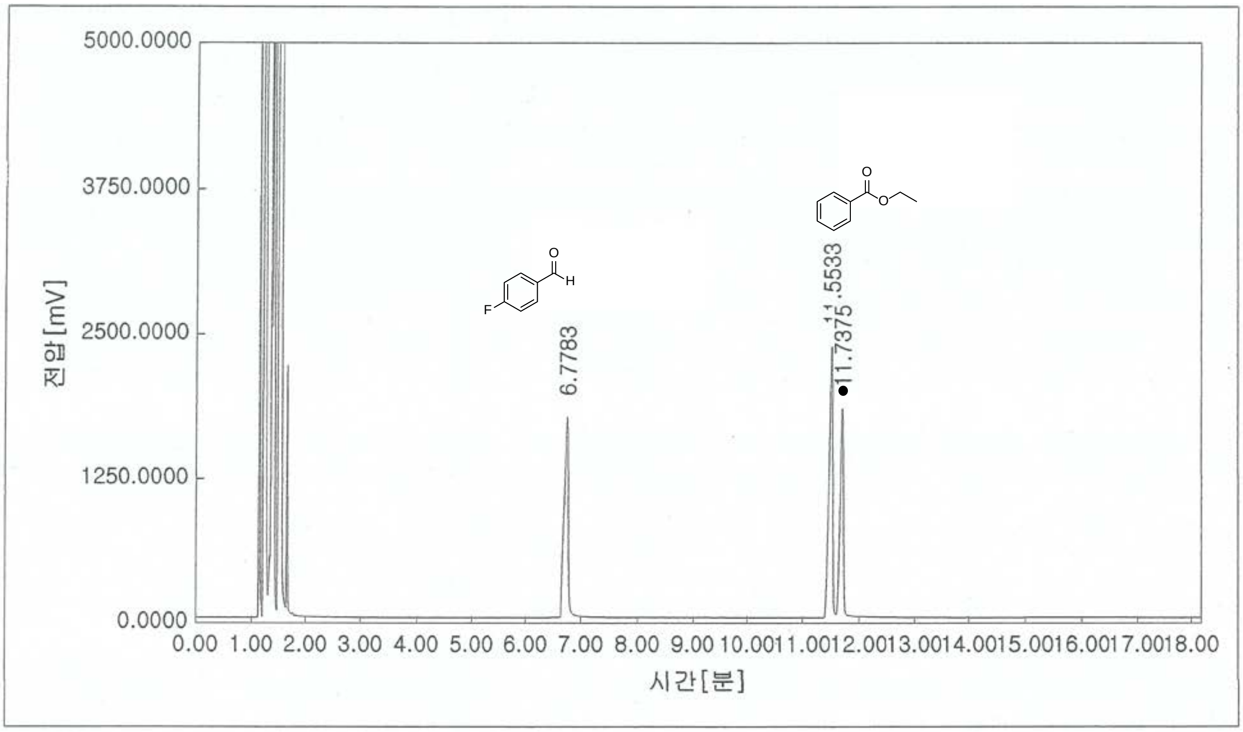

Figure S20: GC chromatogram of entry 1 in Table 2 and (●) represent internal standard.

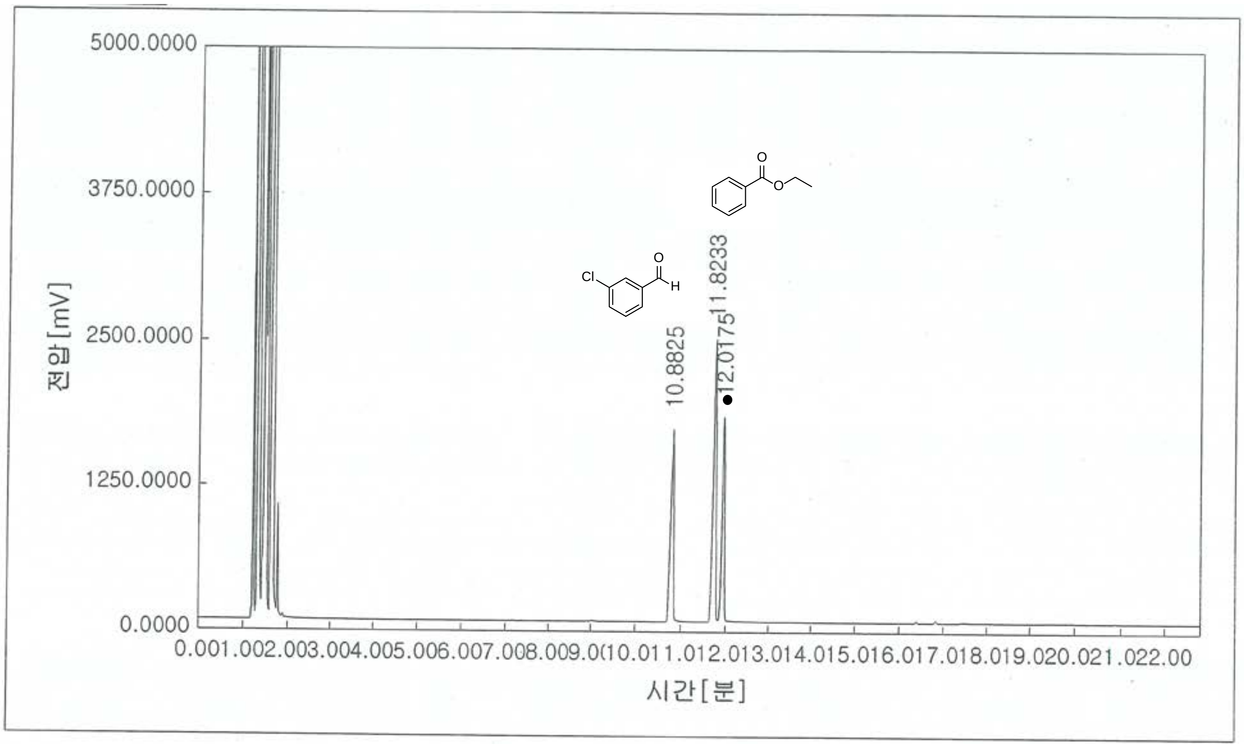

Figure S21: GC chromatogram of entry 2 in Table 2 and **(●)** represent internal standard.

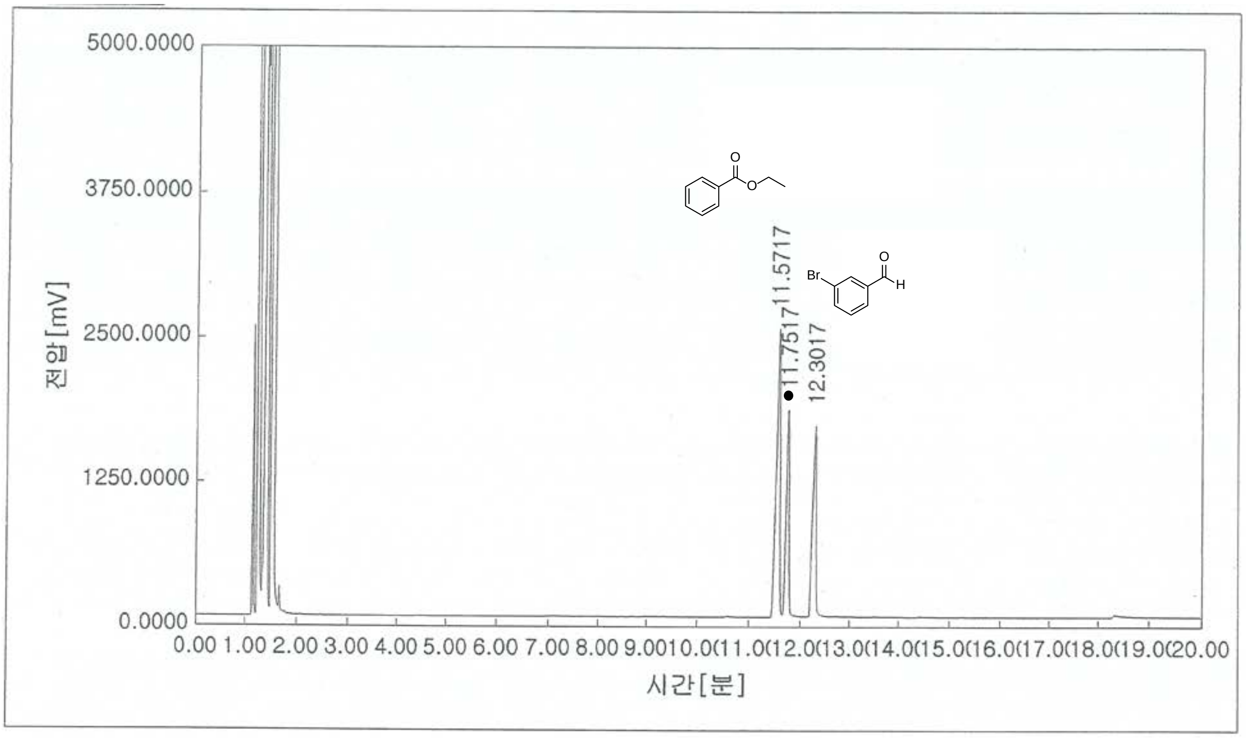

Figure S22: GC chromatogram of entry 3 in Table 2 and **(●)** represent internal standard.

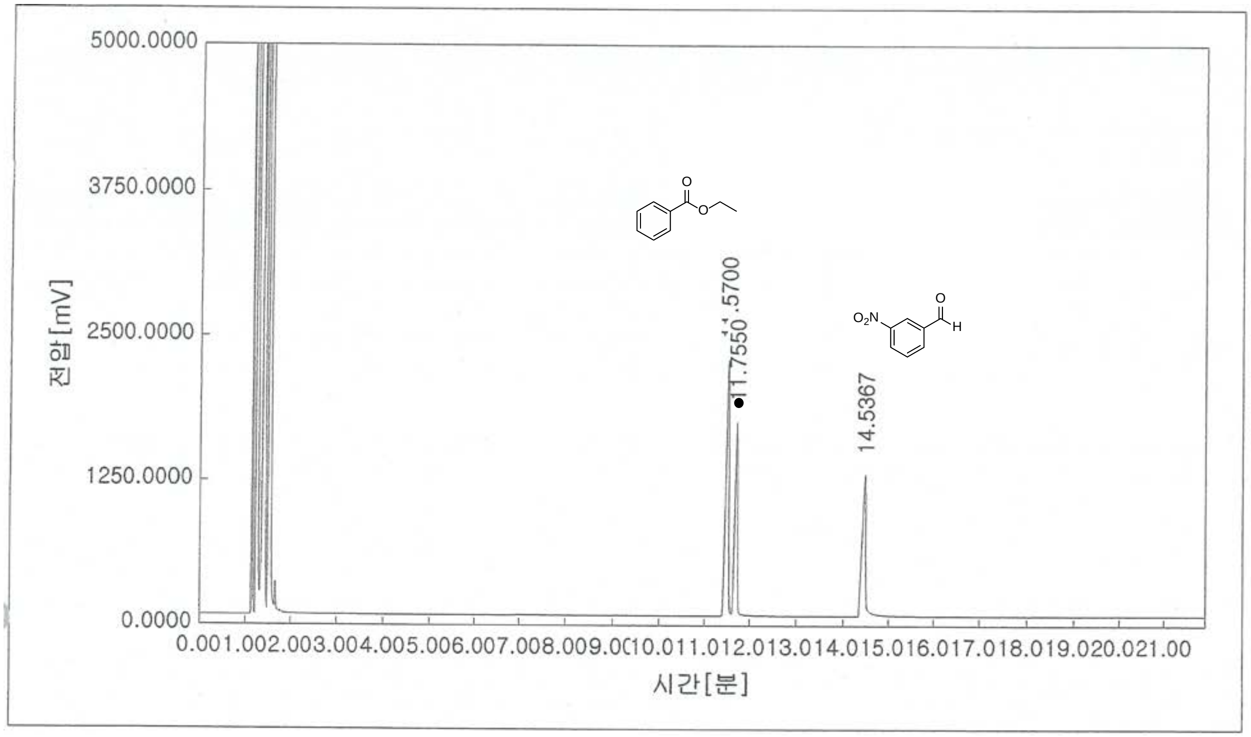

Figure S23: GC chromatogram of entry 4 in Table 2 and (●) represent internal standard.

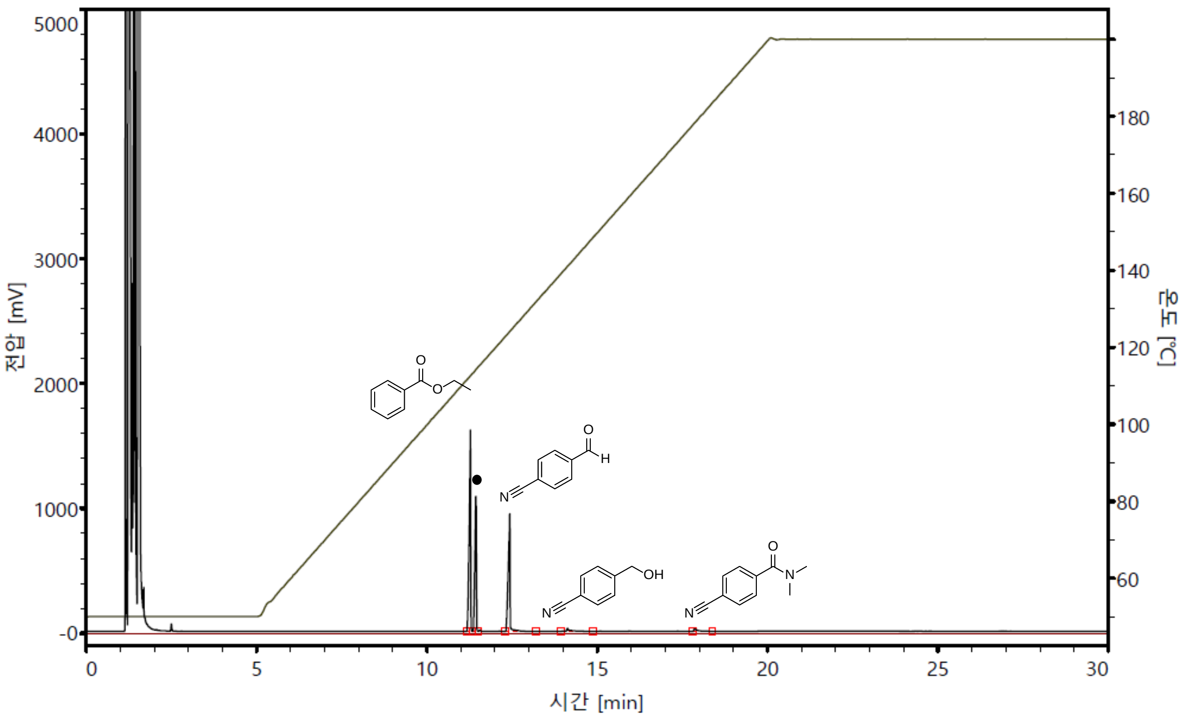

Figure S24: GC chromatogram of entry 5 in Table 2 and (●) represent internal standard.

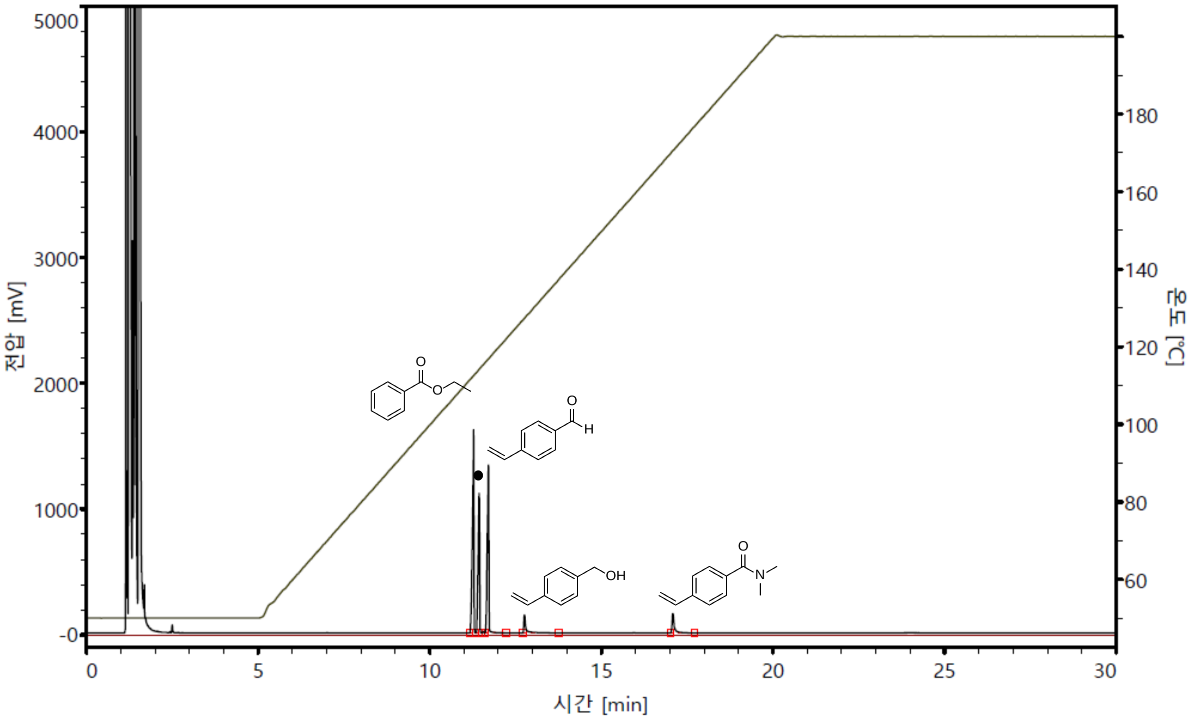

Figure S25: GC chromatogram of entry 6 in Table 2 and (●) represent internal standard.

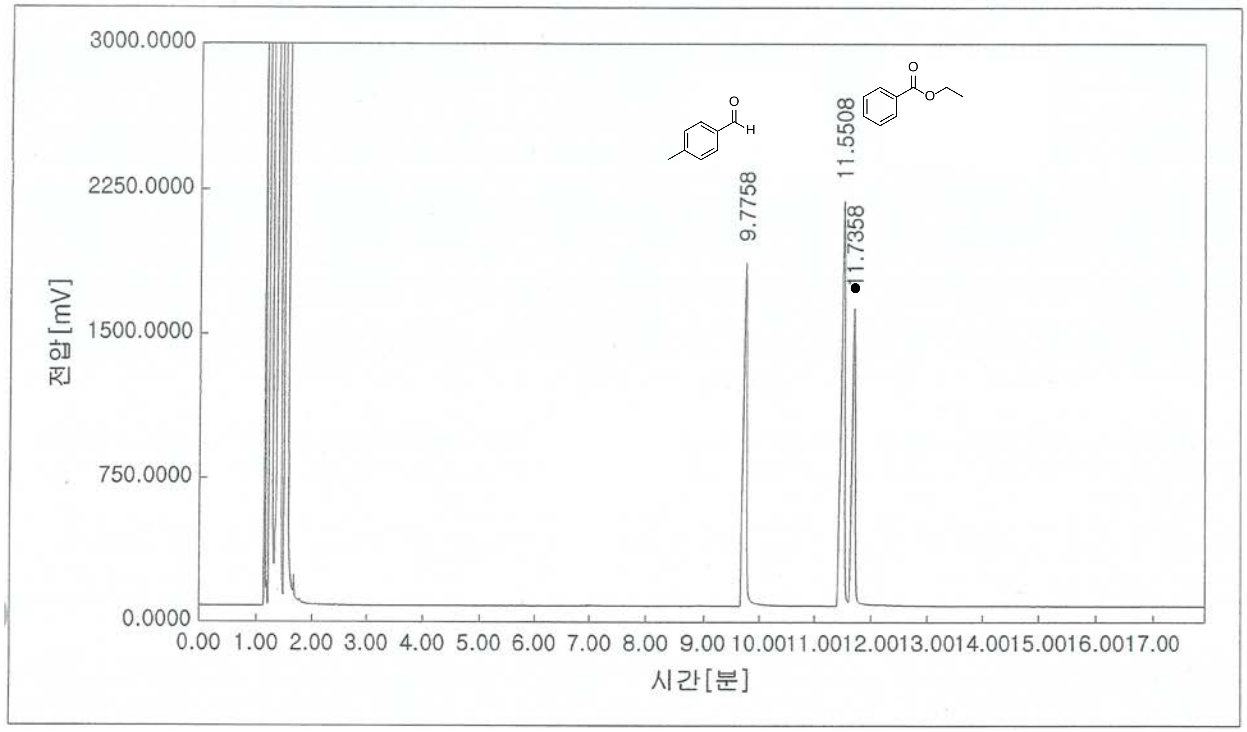

Figure S26: GC chromatogram of entry 7 in Table 2 and (●) represent internal standard.

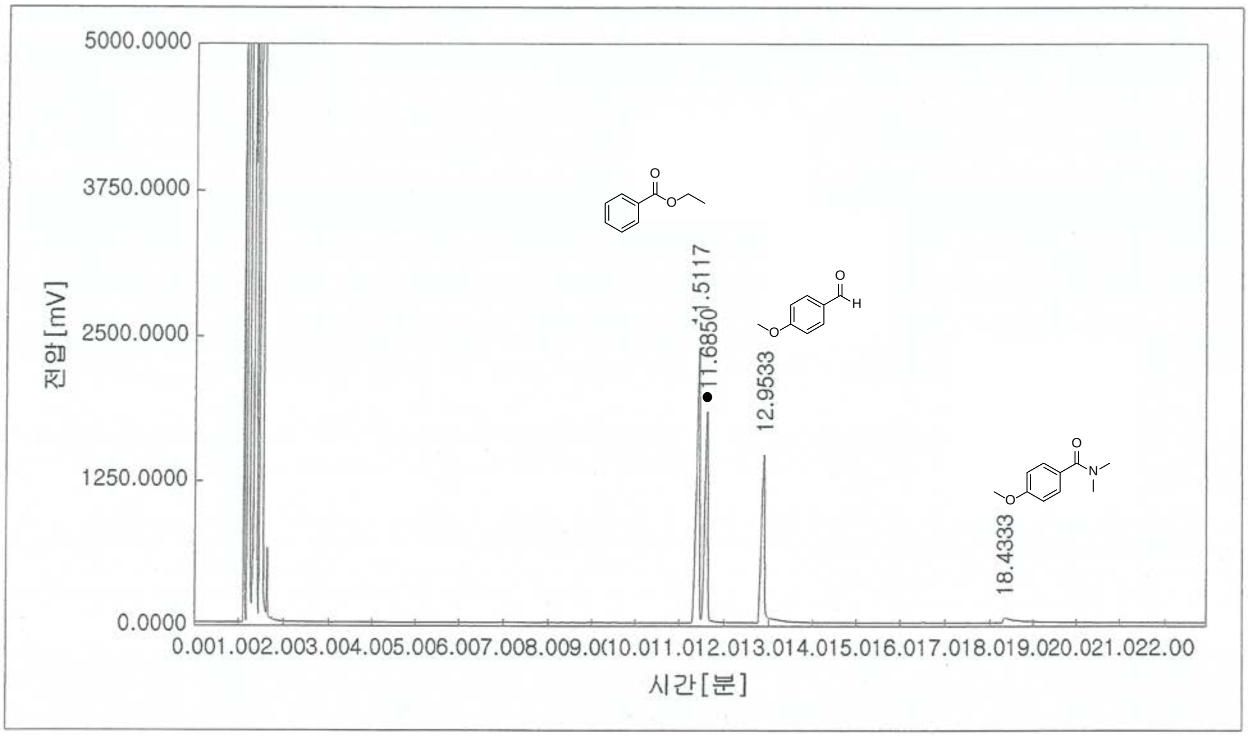

Figure S27: GC chromatogram of entry 8 in Table 2 and **(●)** represent internal standard.

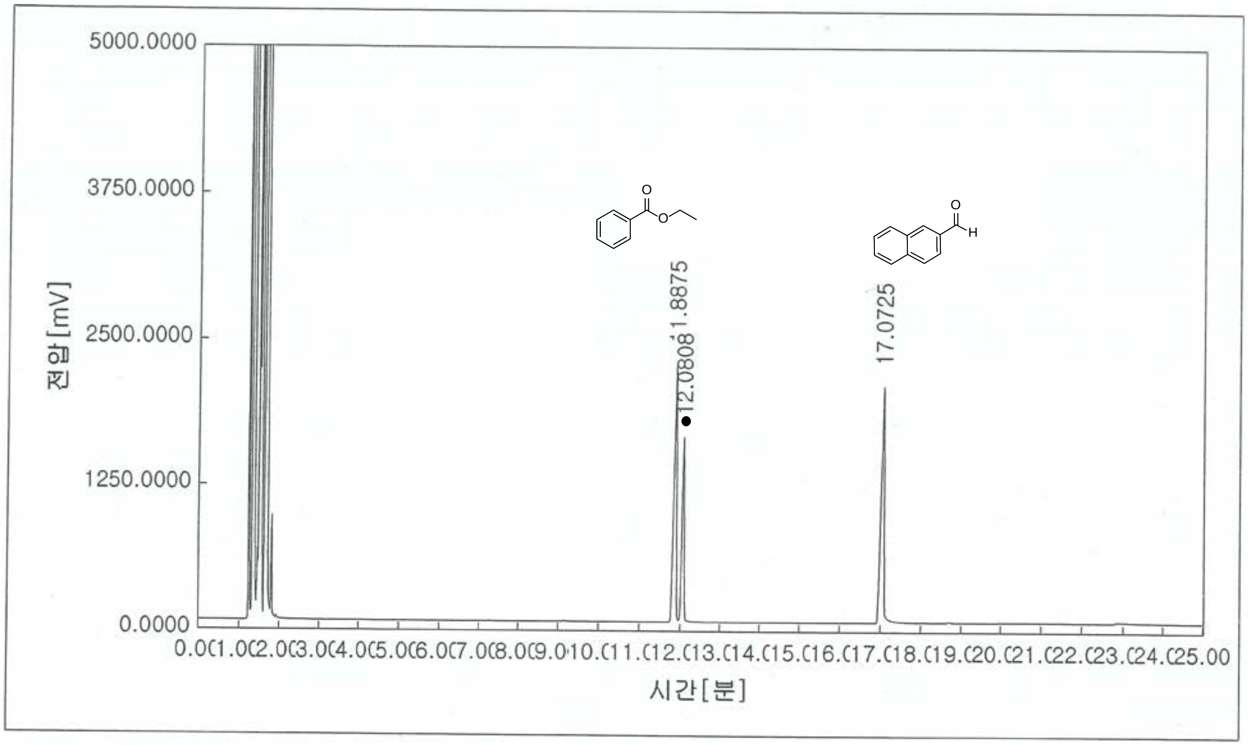

Figure S28: GC chromatogram of entry 9 in Table 2 and **(●)** represent internal standard.

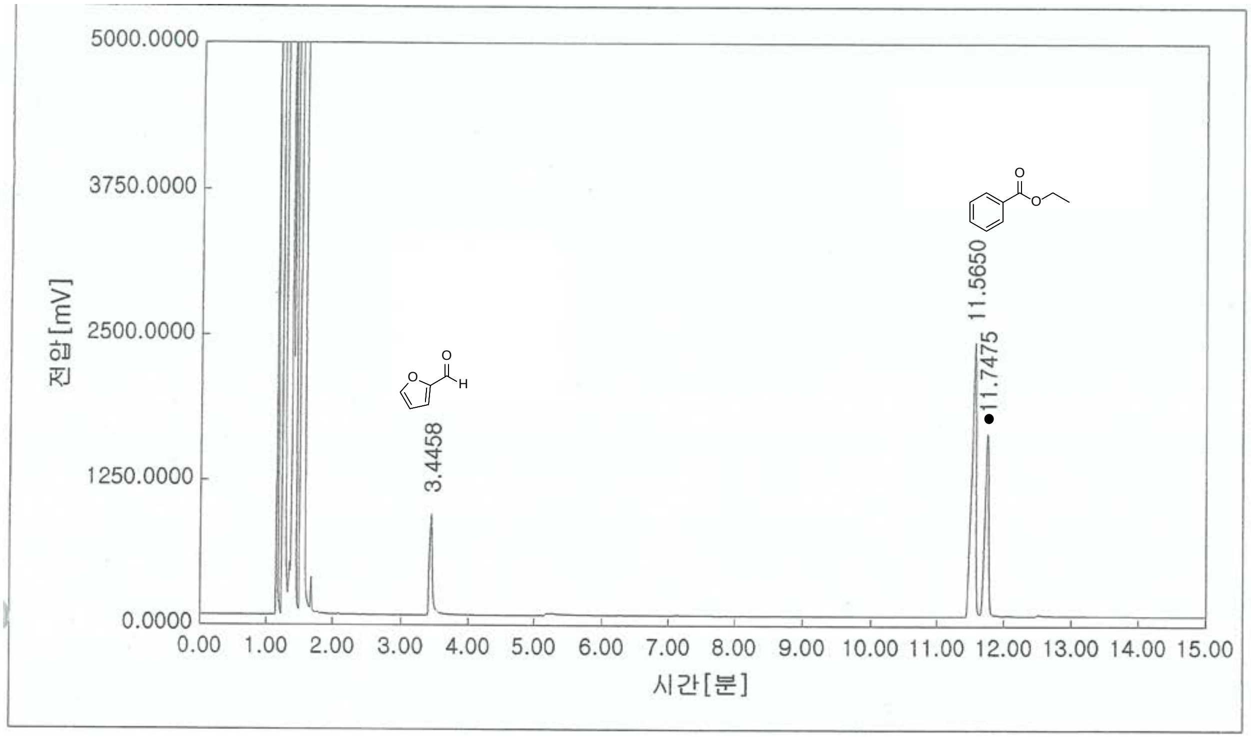

Figure S29: GC chromatogram of entry 10 in Table 2 and (●) represent internal standard.

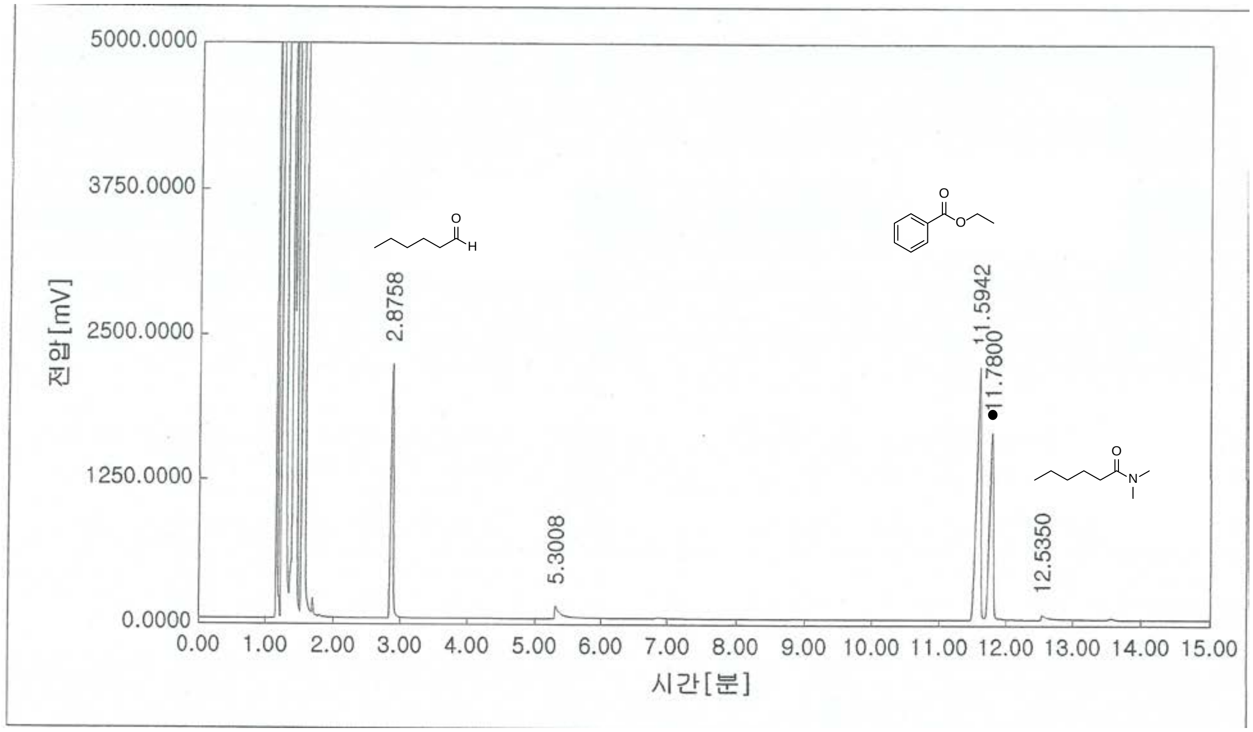

Figure S30: GC chromatogram of entry 11 in Table 2 and (●) represent internal standard.

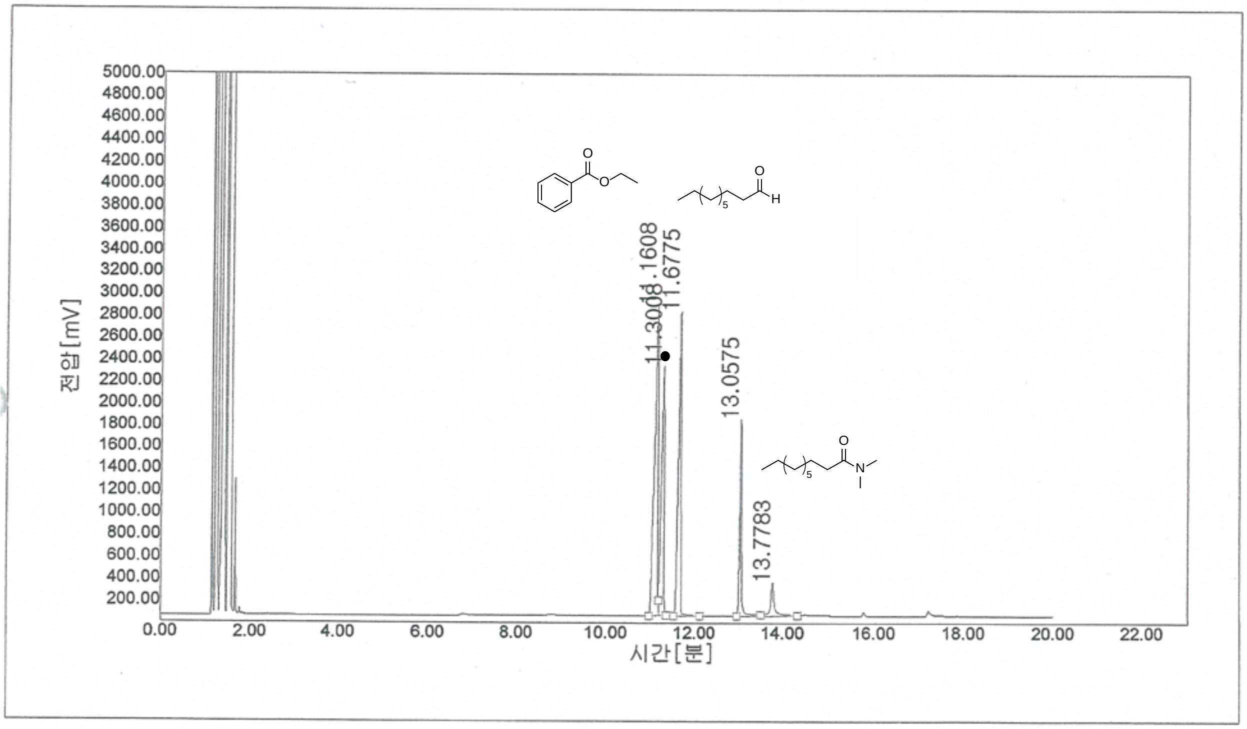

Figure S31: GC chromatogram of entry 12 in Table 2 and (●) represent internal standard.

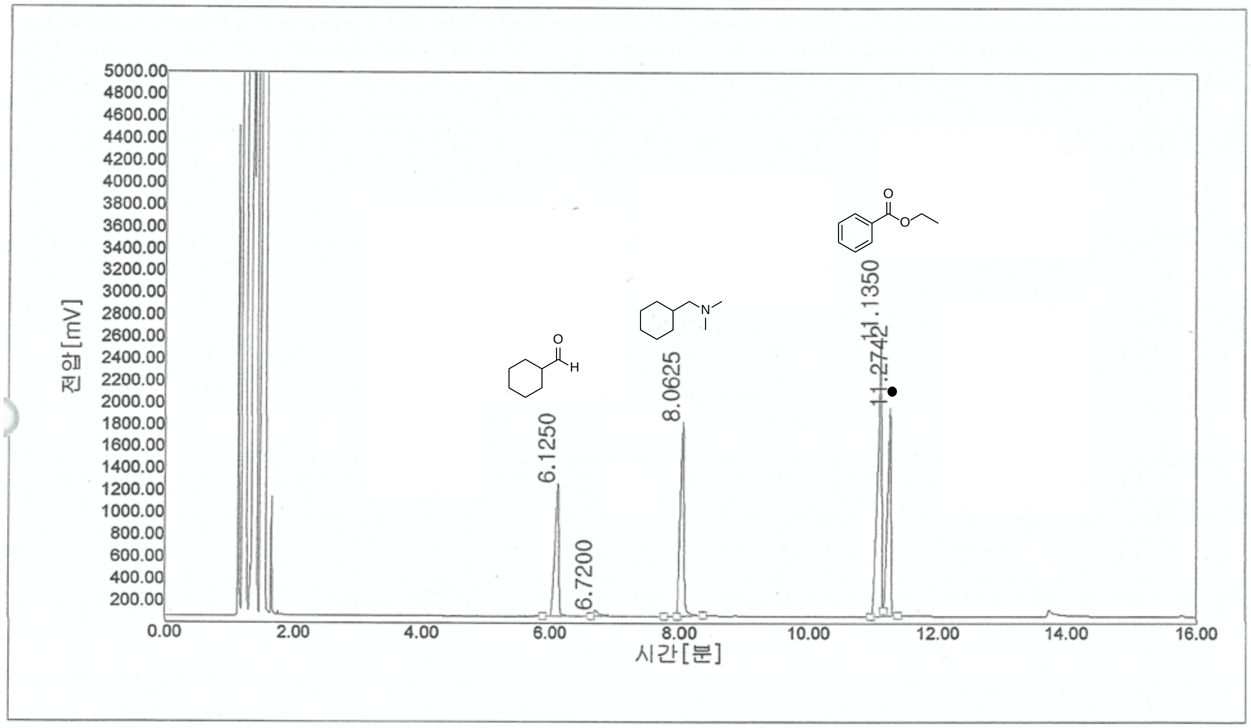

Figure S32: GC chromatogram of entry 13 in Table 2 and (●) represent internal standard.

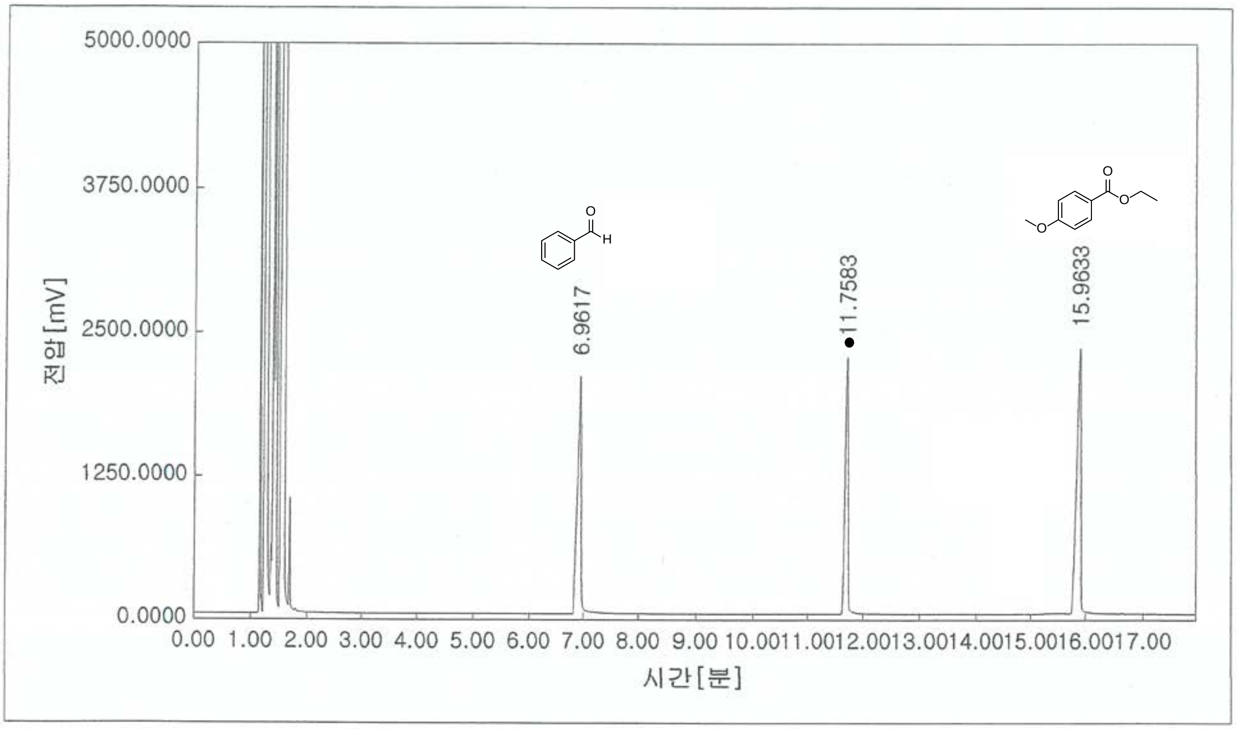

Figure S33: GC chromatogram of entry 1 in Table 3 and **(●)** represent internal standard.

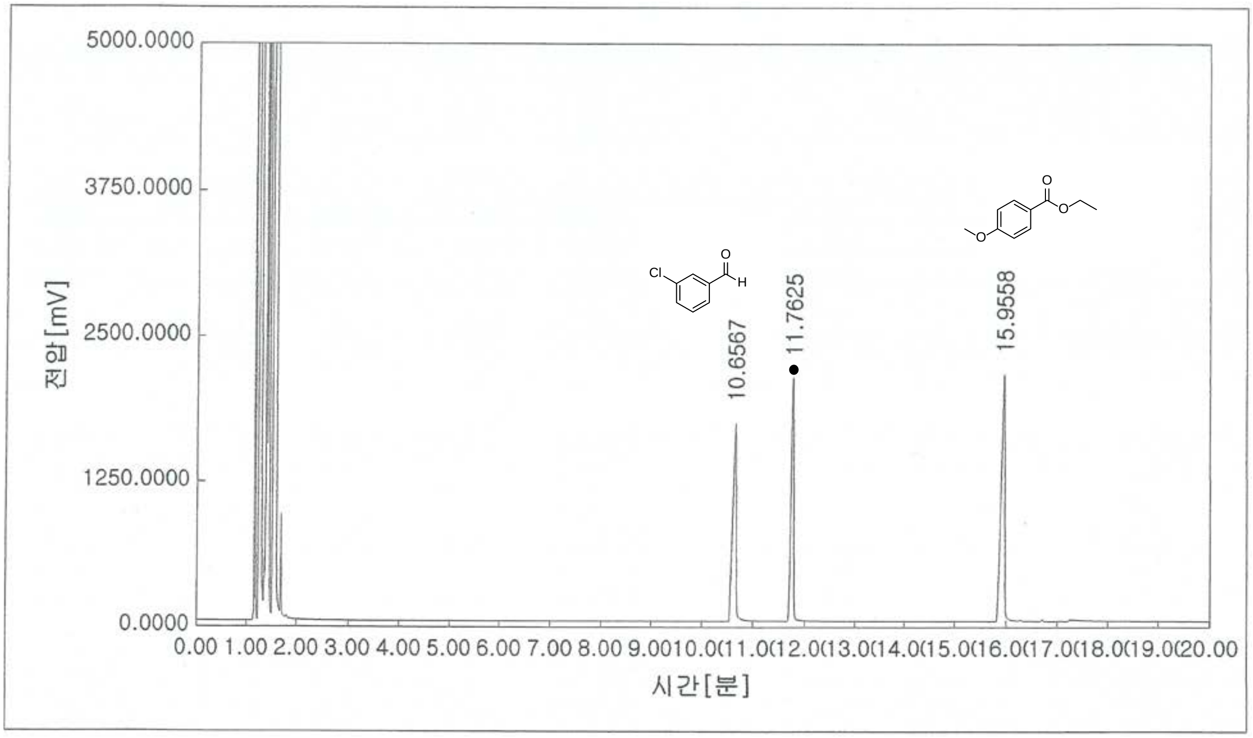

Figure S34: GC chromatogram of entry 2 in Table 3 and **(●)** represent internal standard.

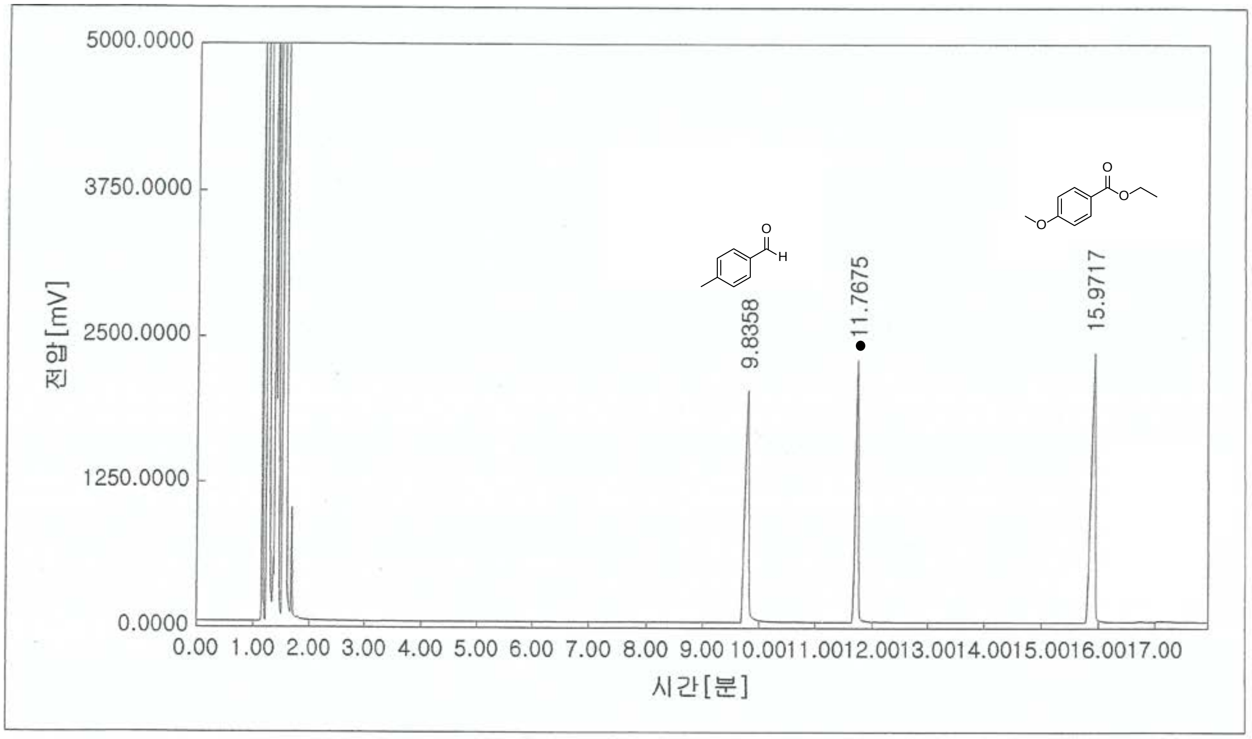

Figure S35: GC chromatogram of entry 3 in Table 3 and **(●)** represent internal standard.

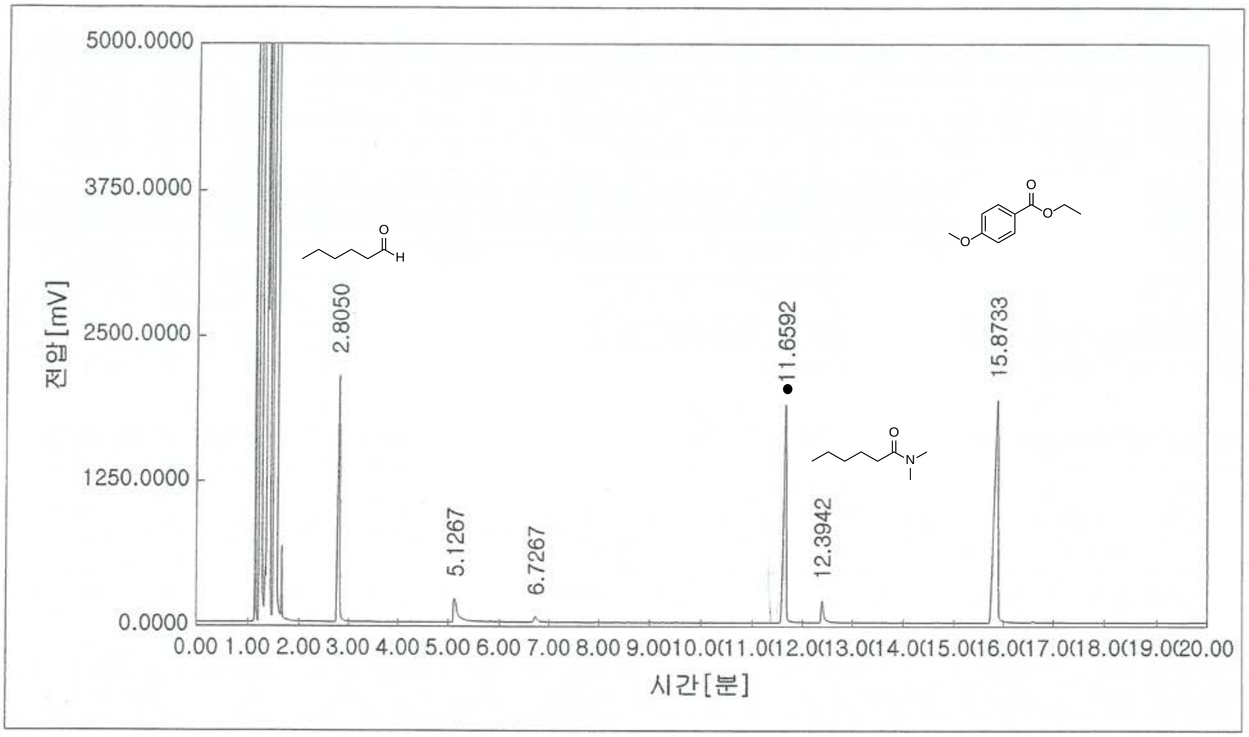

Figure S36: GC chromatogram of entry 4 in Table 3 and **(●)** represent internal standard.

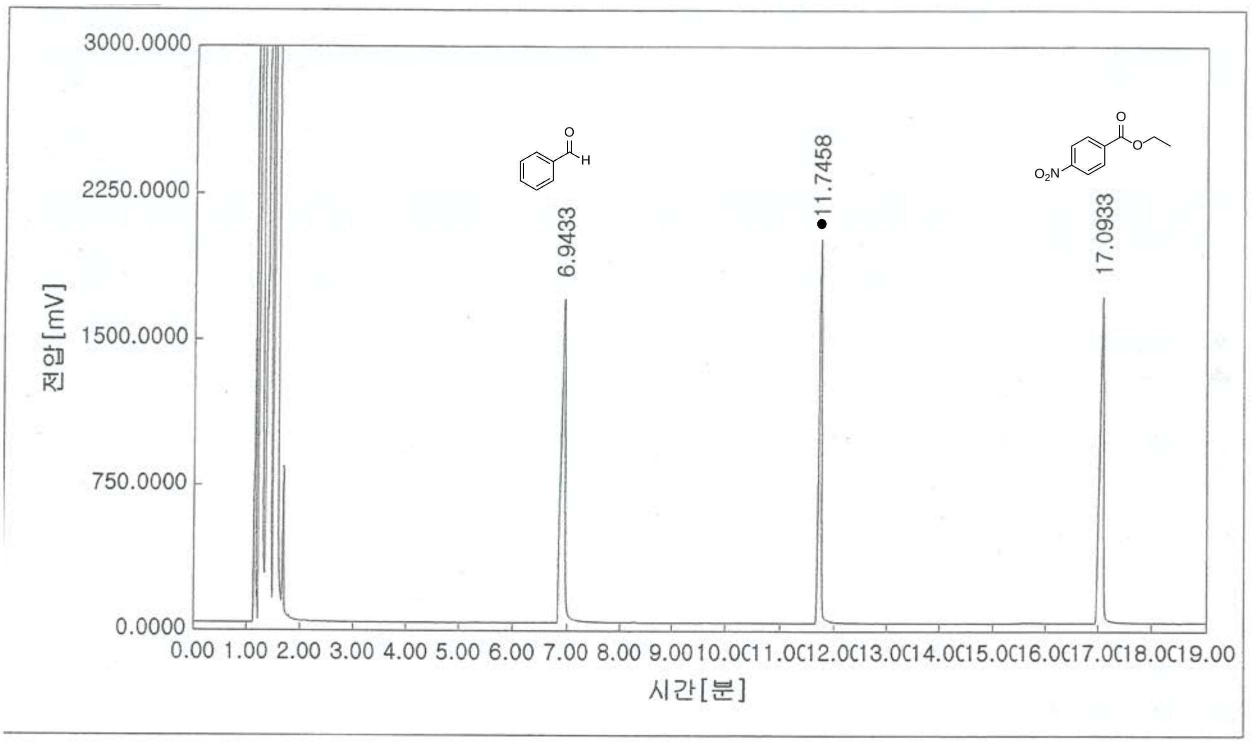

Figure S37: GC chromatogram of entry 5 in Table 3 and **(●)** represent internal standard.

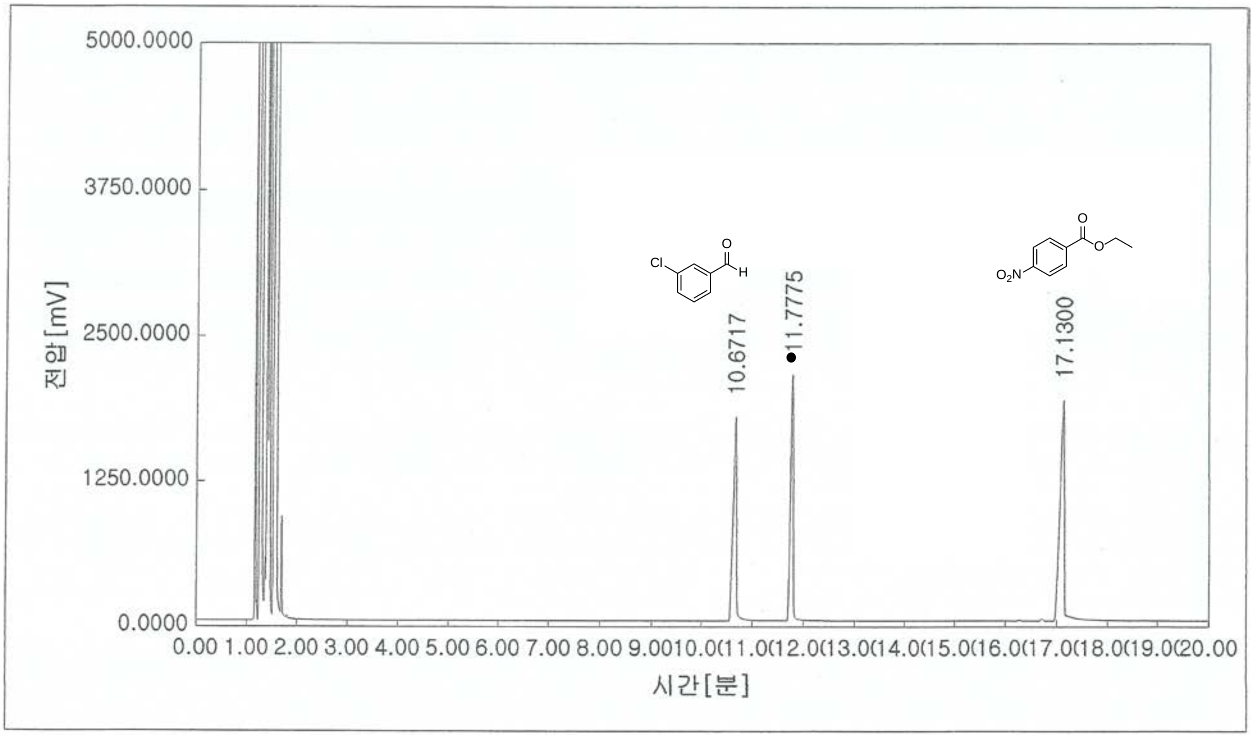

Figure S38: GC chromatogram of entry 6 in Table 3 and **(●)** represent internal standard.

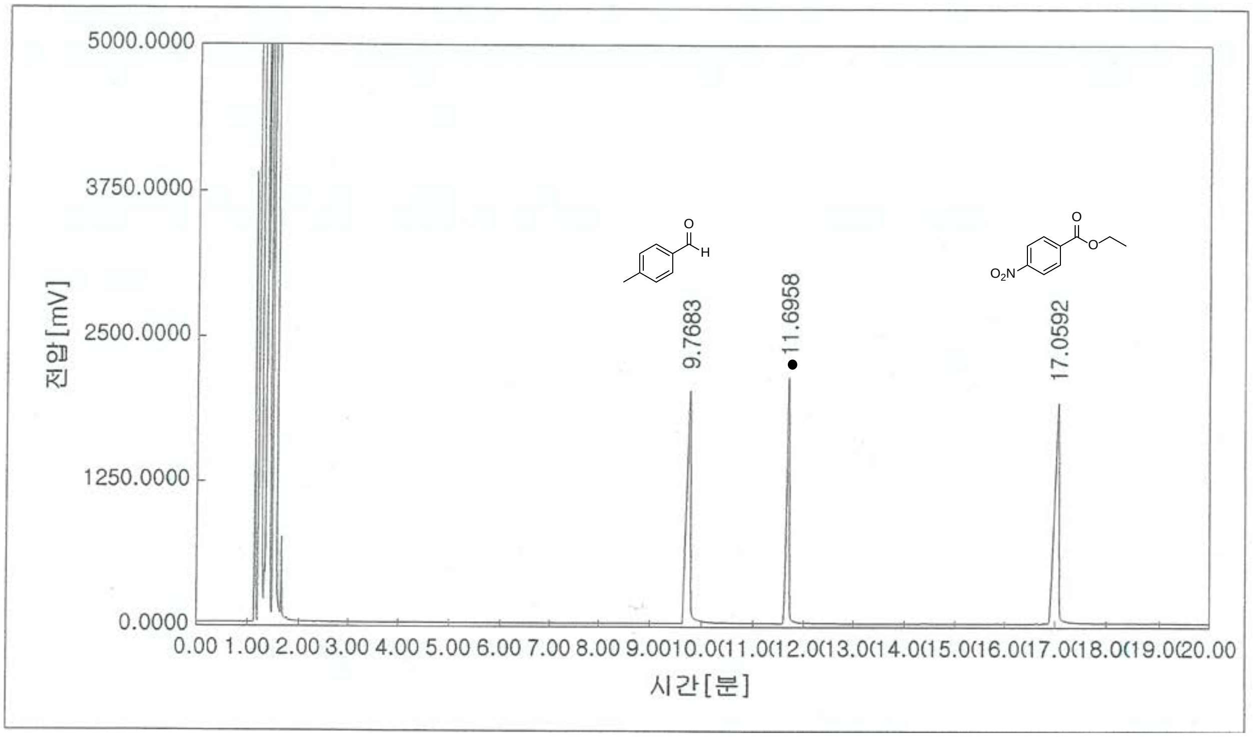

Figure S39: GC chromatogram of entry 7 in Table 3 and **(●)** represent internal standard.

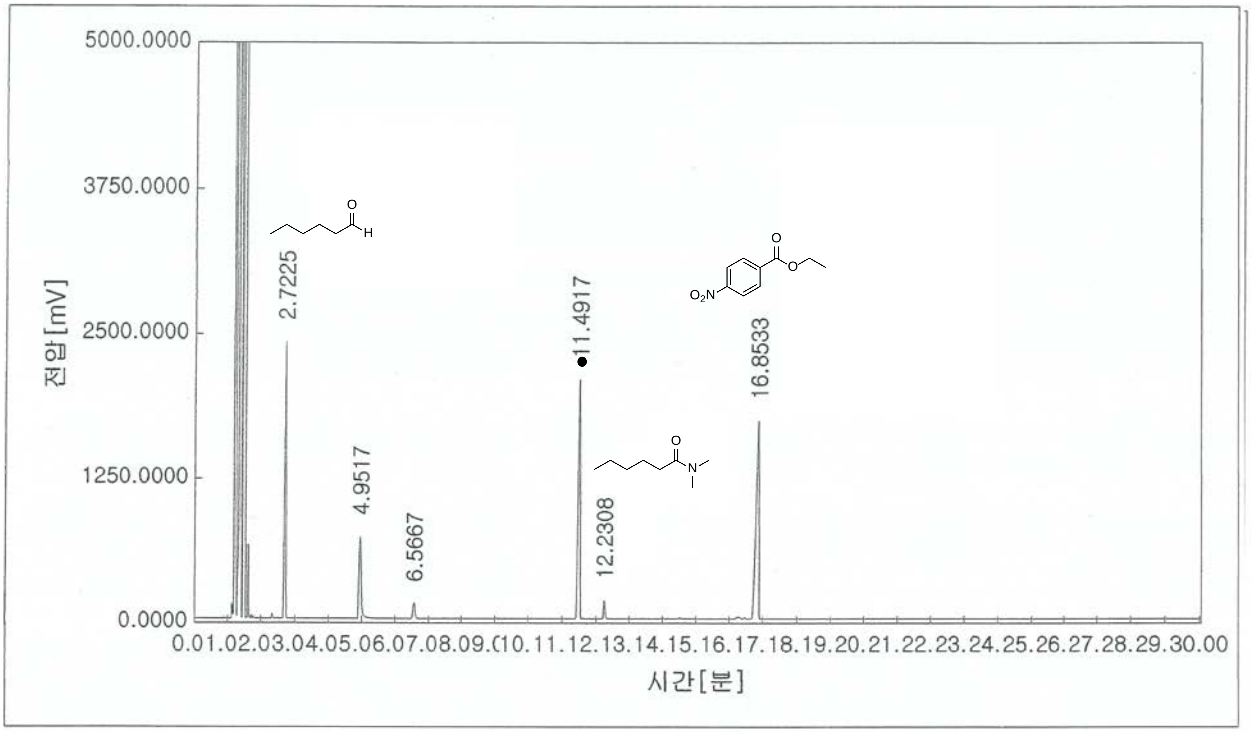

Figure S40: GC chromatogram of entry 8 in Table 3 and **(●)** represent internal standard.

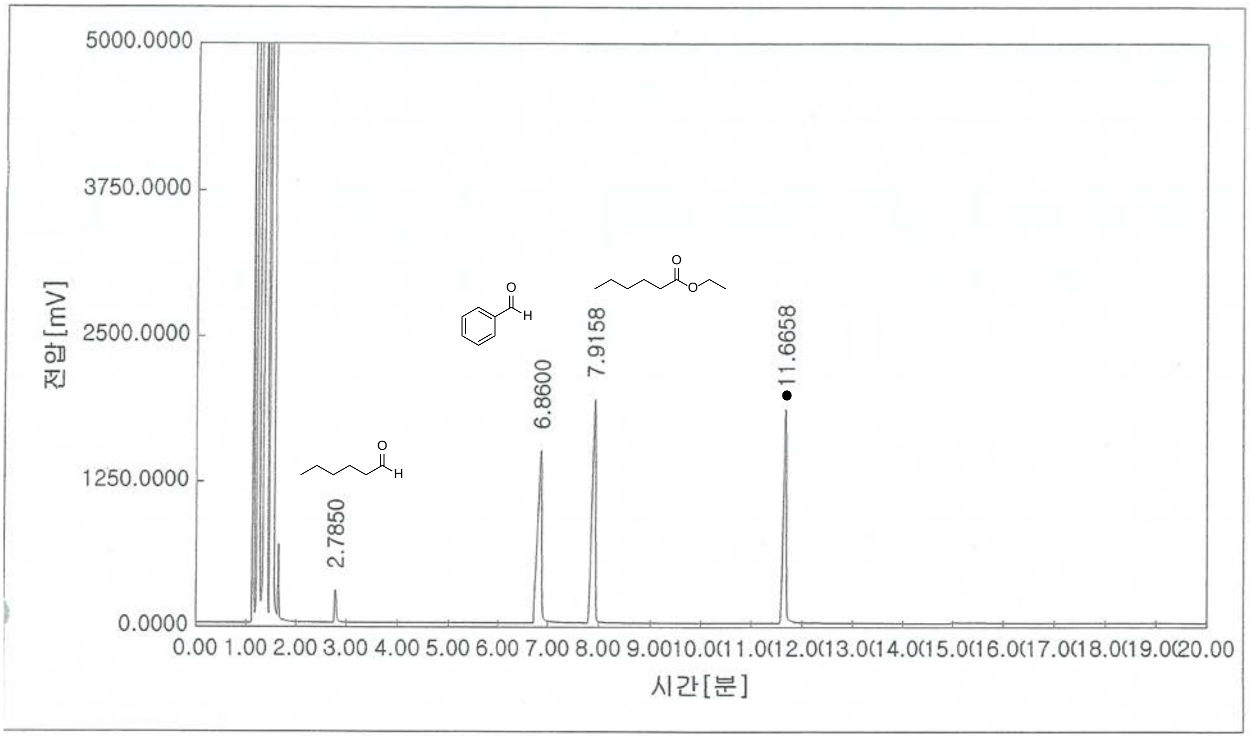

Figure S41: GC chromatogram of entry 9 in Table 3 and **(●)** represent internal standard.

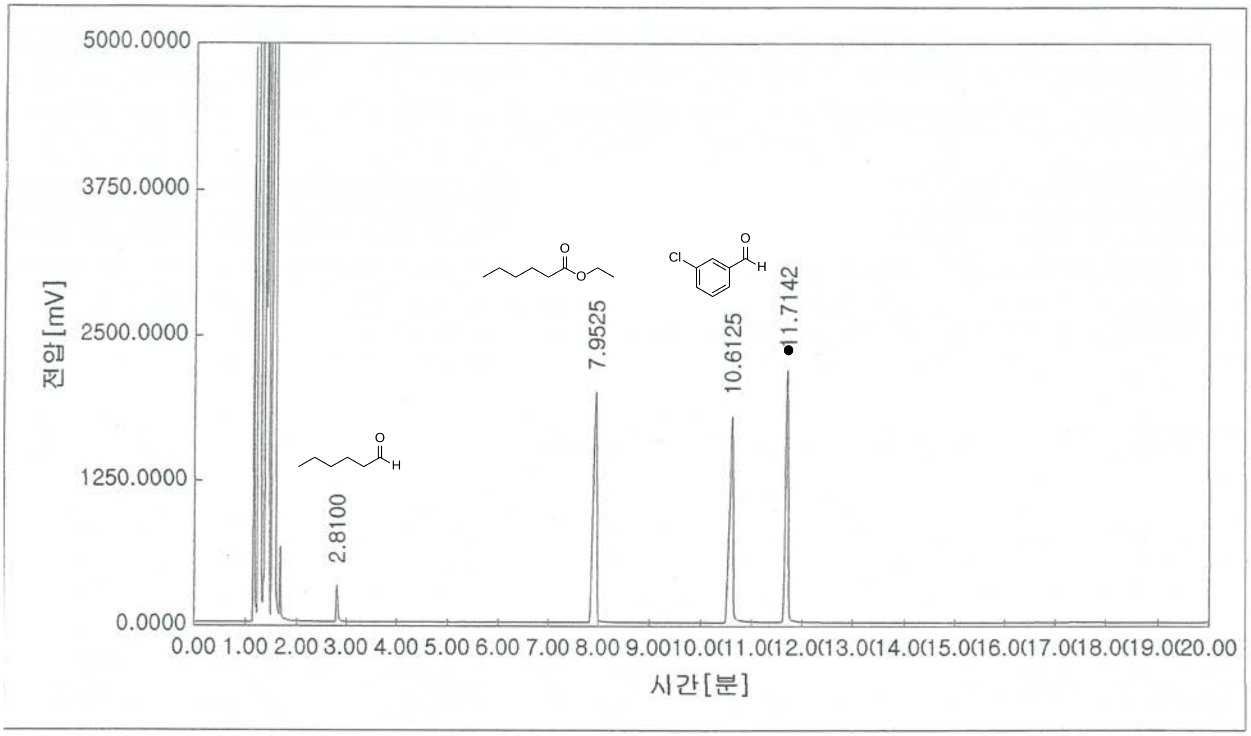

Figure S42: GC chromatogram of entry 10 in Table 3 and **(●)** represent internal standard.

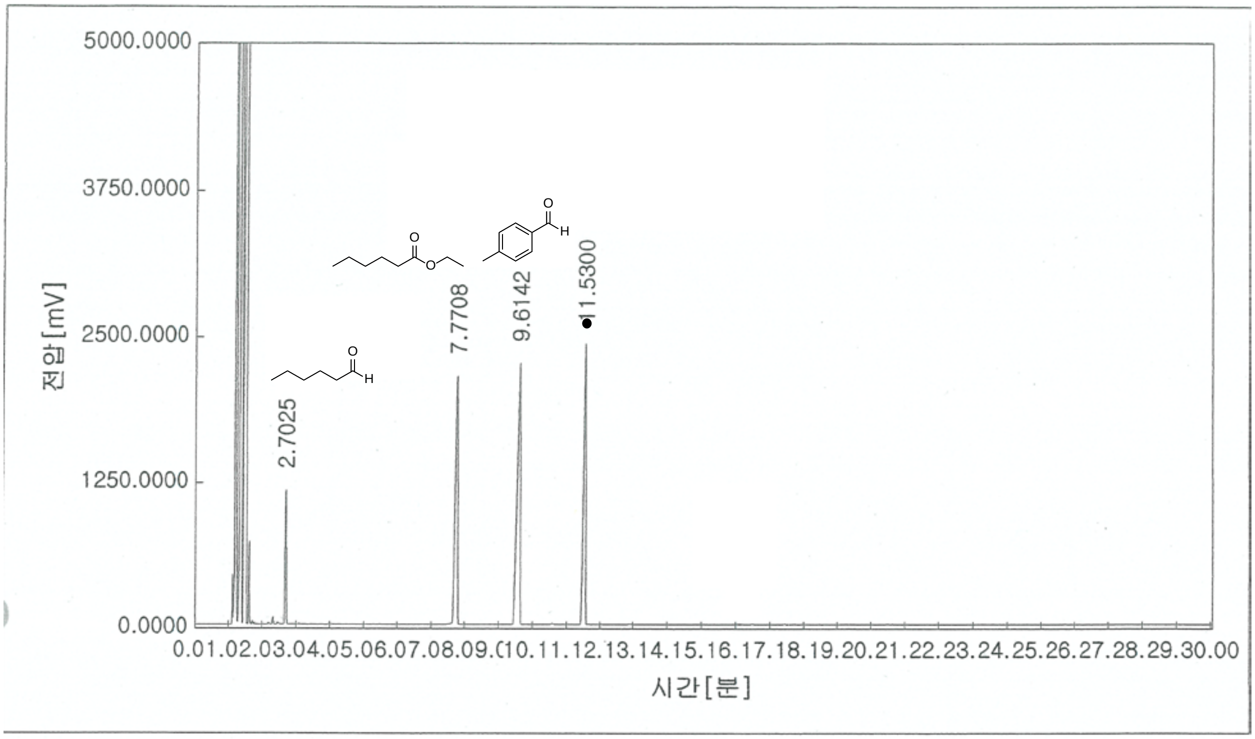

Figure S43: GC chromatogram of entry 11 in Table 3 and **(●)** represent internal standard.

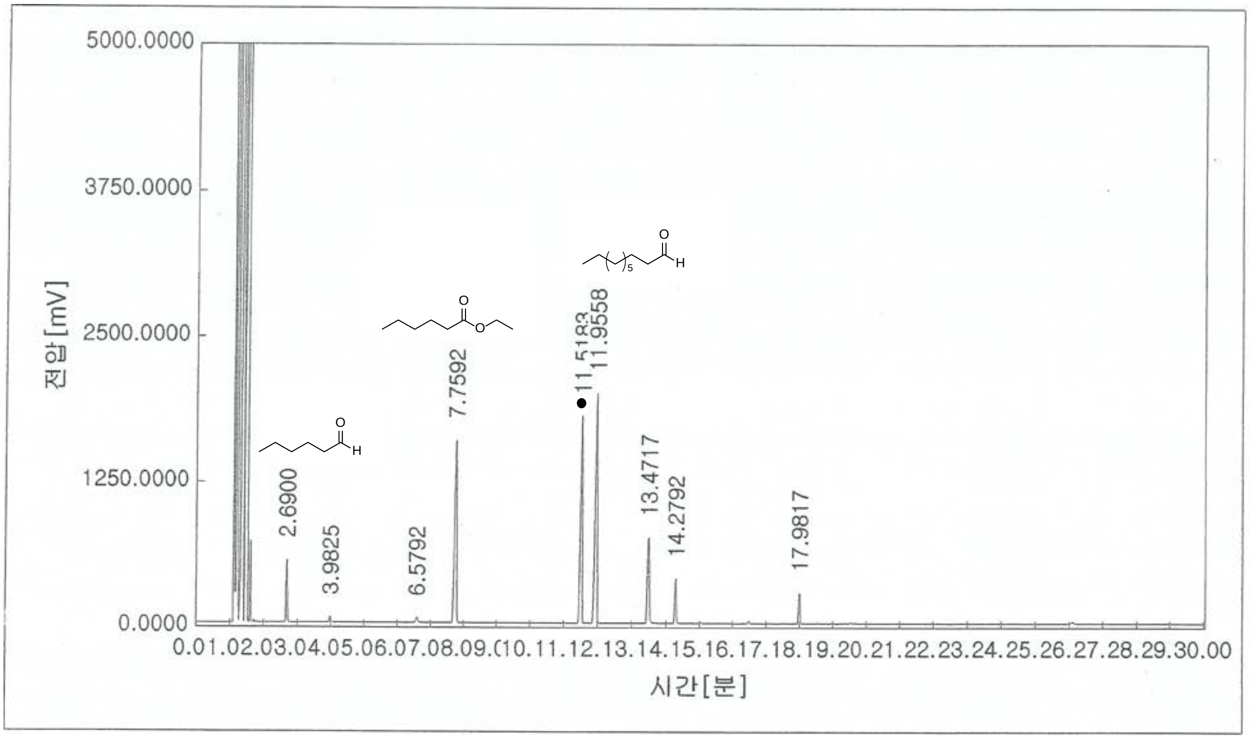

Figure S44: GC chromatogram of entry 12 in Table 3 and **(●)** represent internal standard.

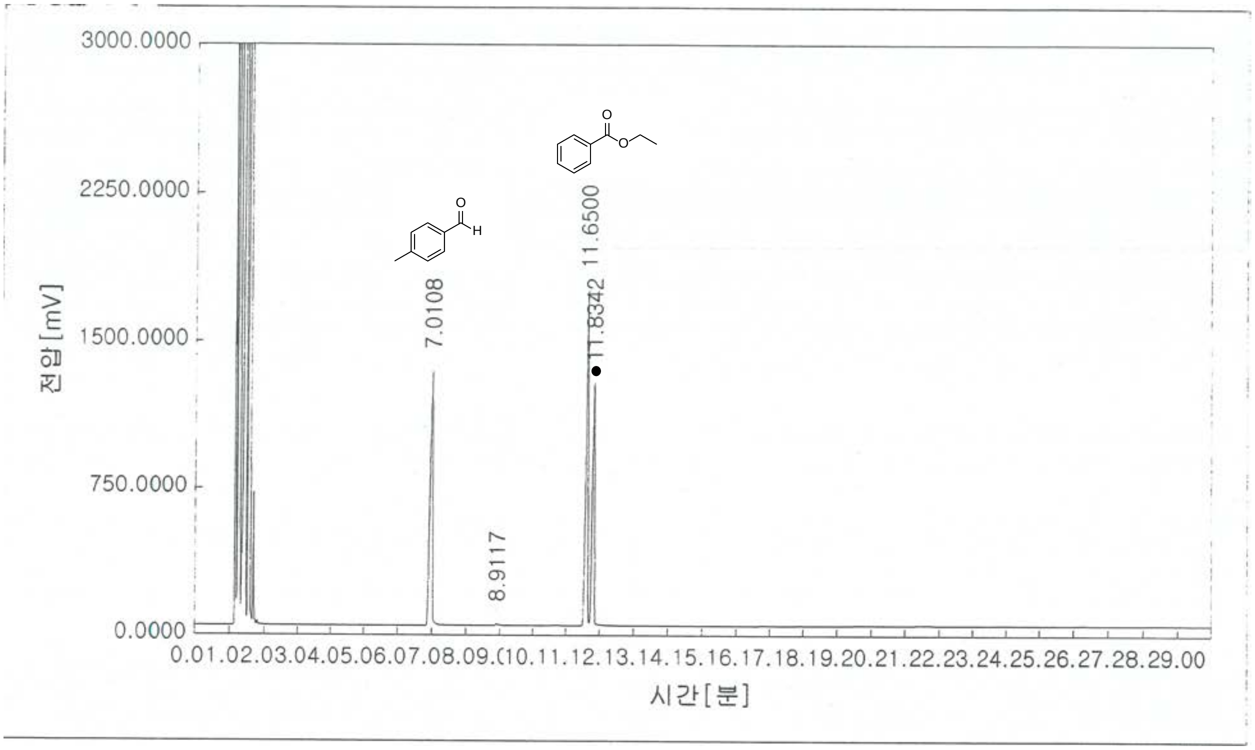

Figure S45: GC chromatogram of entry 1 in Table 4 and (●) represent internal standard.

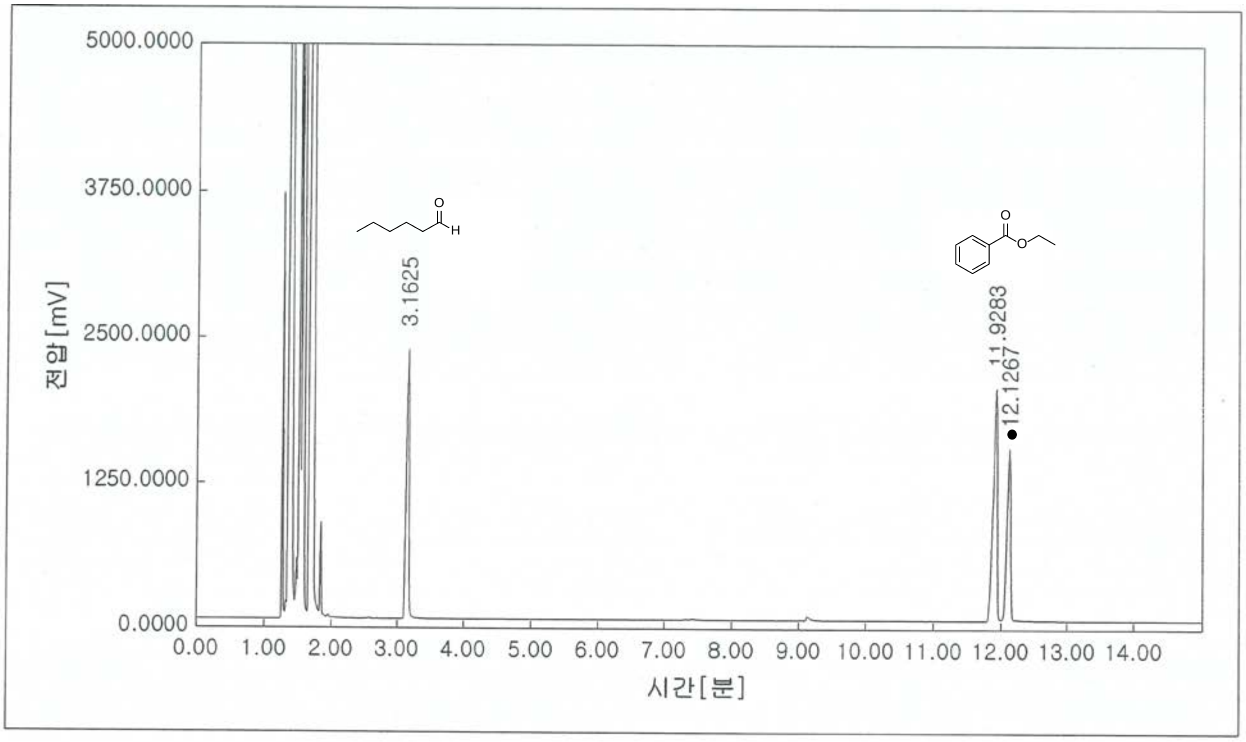

Figure S46: GC chromatogram of entry 2 in Table 4 and (●) represent internal standard.

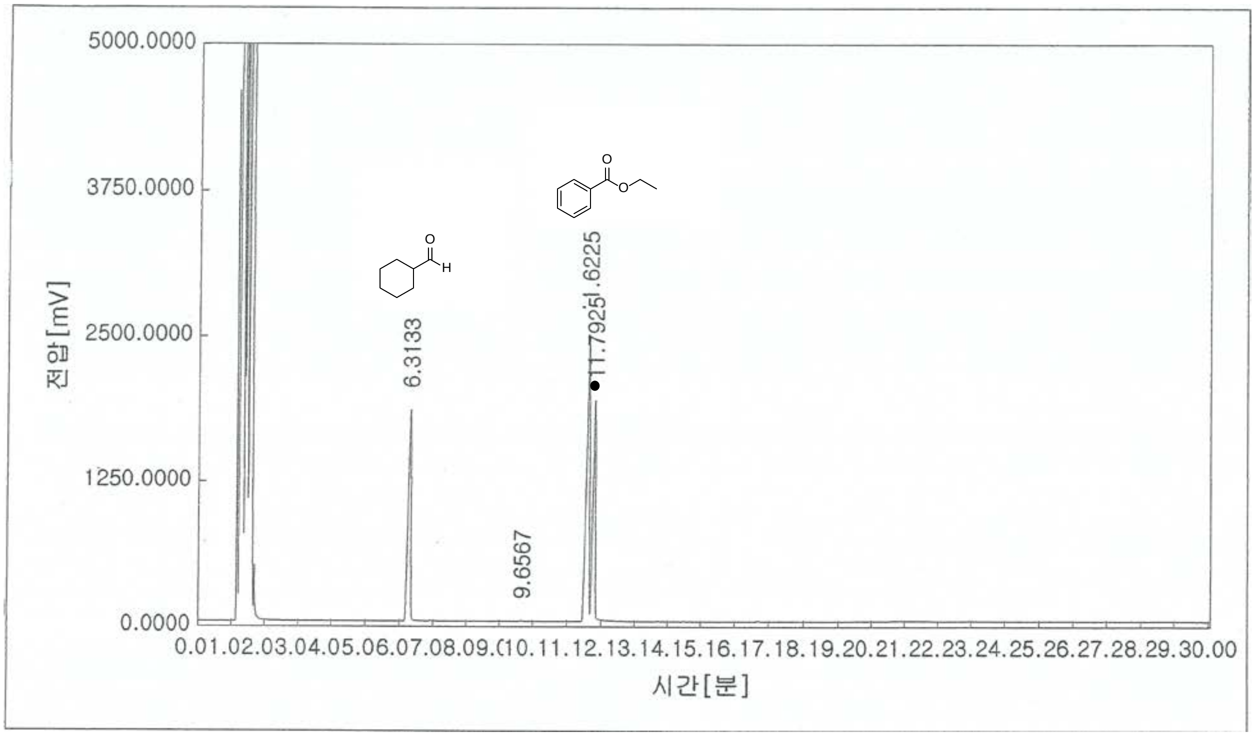

Figure S47: GC chromatogram of entry 3 in Table 4 and **(●)** represent internal standard.

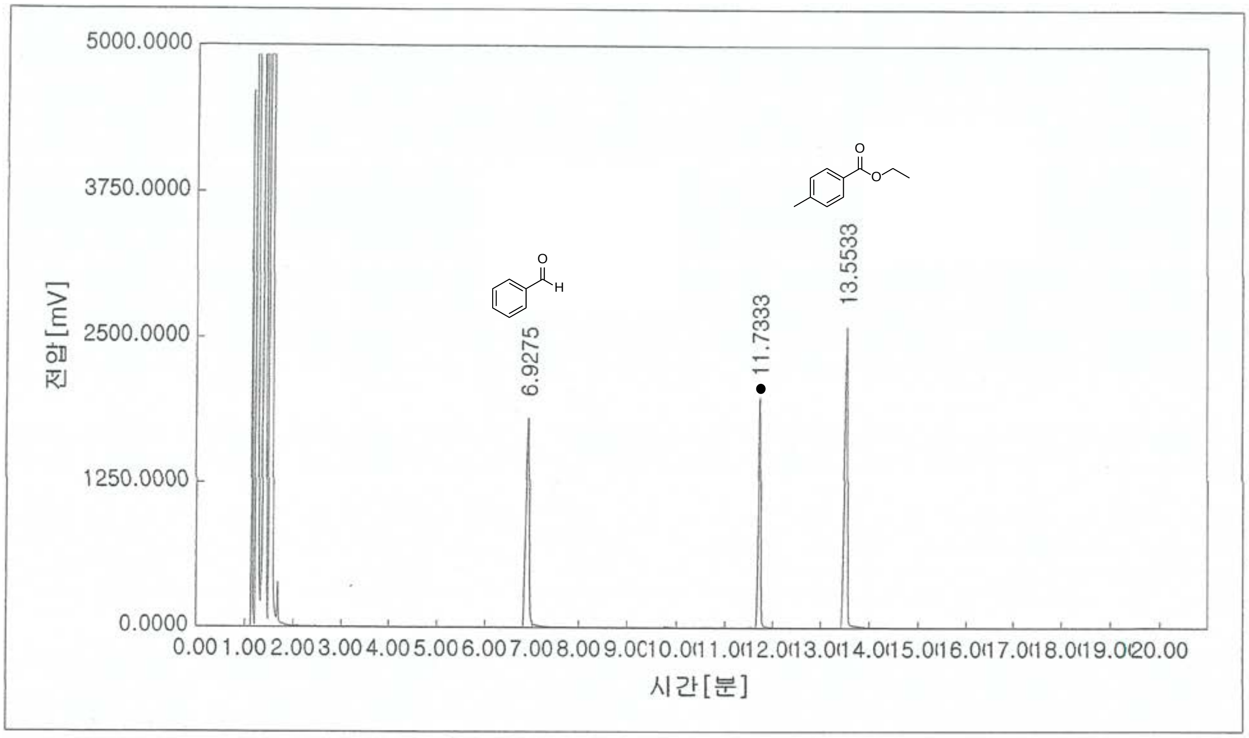

Figure S48: GC chromatogram of entry 4 in Table 4 and **(●)** represent internal standard.

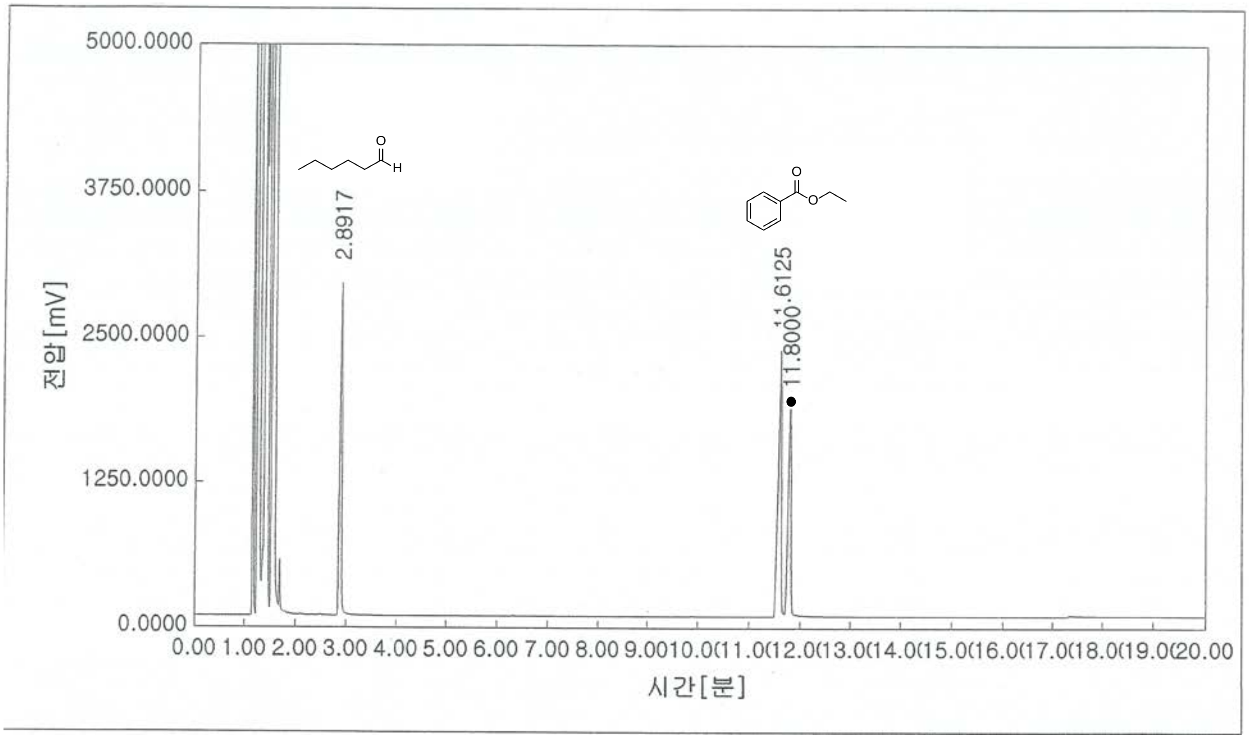

Figure S49: GC chromatogram of entry 5 in Table 4 and **(●)** represent internal standard.

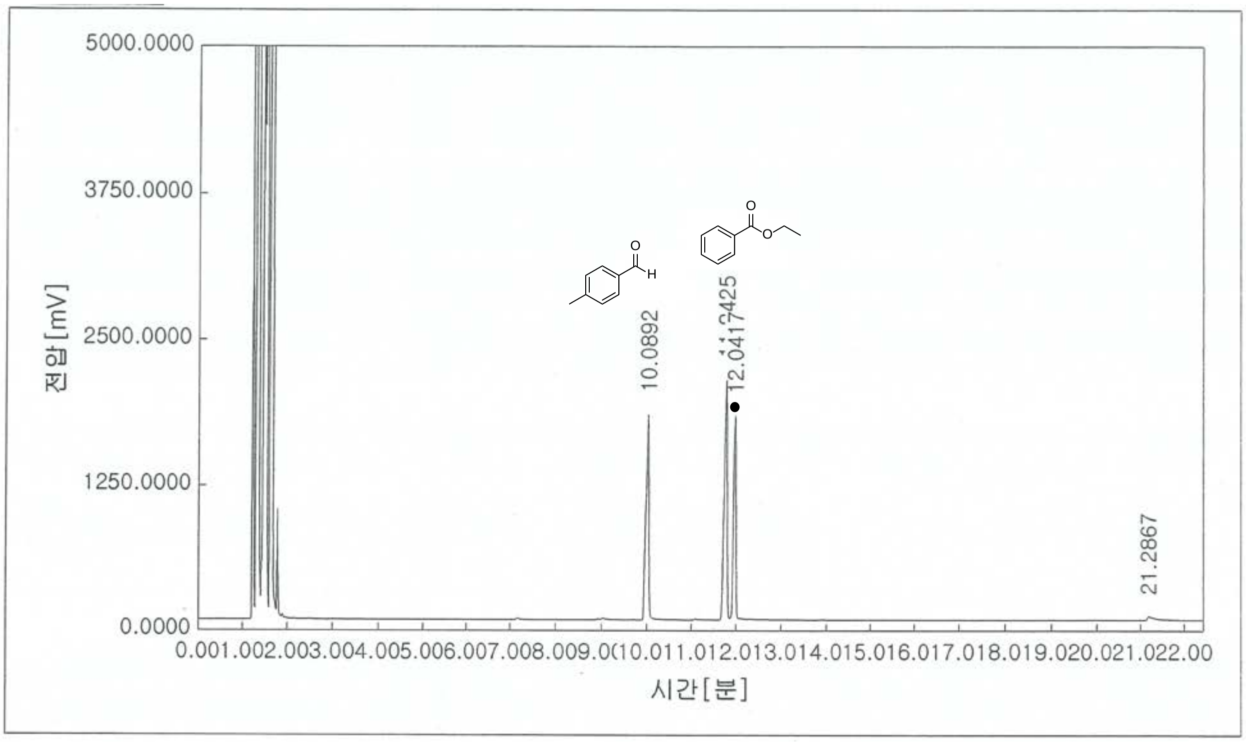

Figure S50: GC chromatogram of entry 6 in Table 4 and **(●)** represent internal standard.

References:

S1) N. Iranpoor, F. Panahi, F. Roozbin, S. Erfan and S. Rahimi, *J. Org. Chem*, **2016**, *9*, 1781.

S2) F. Mäsing, A. Mardyukov, C. Doerenkamp, H. Eckert, U. Malkus, H. Nüsse, J. Klingauf, A. Studer, *Angew. Chem. Int. Ed.* **2015**, *54*, 12612.

S3) Z. Liu, J. Zhang, S. Chen, E. Shi, Y. Xu and X. Wan, *Angew. Chem. Int. Ed*. **2012**, *51*, 3231.

S4) M. Sayes, A. B. Charette, *Green Chem*. **2017**, *19*, 5060.

S5) M. M. Faul, A. M. Ratz, K. A. Sullivan, W. G. Trankle, L. L. Winneroski, *J. Org. Chem*. **2001**, *66*, 5772.

S6) C. W. Cheung, M. L. Ploeger, X. Hu, *Nat. Commun*. **2017**, *8*, 14878.

S7) S. Imai, H. Togo, *Tetrahedron* **2016**, *72*, 6948.

S8) H. Tsuji, H. Yamamoto, *J. Am. Chem. Soc*. **2016**, *138*, 14218.
